# miR147 promotes mucosal integrity and healing in intestinal inflammation

**DOI:** 10.1172/jci.insight.190466

**Published:** 2025-09-16

**Authors:** Agnieszka K. Czopik, Arash Dabiri, Chia-Hao Tung, Victoria Vaughn, Xiangsheng Huang, Jinlian Wang, Hui Li, Nicolas F. Moreno, Natalia V. Piwko, Katherine Figarella, Hongfang Liu, Zhongming Zhao, Xiaoyi Yuan, Holger K. Eltzschig

**Affiliations:** 1 Department of Anesthesiology, Critical Care and Pain Medicine, McGovern Medical School,; 2 Center for Precision Health, McWilliams School of Biomedical Informatics,; 3 Division of Gastroenterology, Hepatology & Nutrition, Department of Internal Medicine,; 4 Department of Health Data Science and AI, McWilliams School of Biomedical Informatics, and; 5 Center for OUTCOMES RESEARCH, University of Texas Health Science Center at Houston, Houston, Texas, USA.

**Keywords:** Gastroenterology, Inflammation, Inflammatory bowel disease

## Abstract

The intestinal mucosal epithelium forms a barrier between luminal contents and the body. MicroRNAs (miRNAs) regulate mucosal homeostasis by controlling inflammatory responses and structural integrity. Here, we discovered a protective role for miR147 in intestinal inflammation using a *miR147tdTomato* reporter mouse. miR147 was enriched in the intestines, with the highest expression in the colonic epithelial cells at the luminal surface, with prominent expression in differentiated enterocytes. Mice with general or intestinal epithelial deletion of *miR147* showed increased intestinal inflammation and diminished mucosal healing during colitis. RNA sequencing of miR147-deficient cells showed dysregulated immune signaling, with upregulated proinflammatory cytokine pathways and reduced type I interferon responses and revealed Ndufa4 as a likely miR147 target. Ndufa4, a mitochondrial protein regulating energy metabolism and inflammation, was elevated at the crypt base, inversely correlating with miR147. Mice lacking the miR147 binding site in Ndufa4’s 3′-UTR phenocopied miR147-deficient mice during colitis. Spatial and single-cell transcriptomic analyses in murine and human colons showed mutually exclusive miR147 and Ndufa4 expression, consistent with a regulatory relationship in epithelial differentiation and metabolism. These findings underscore miR147’s role in intestinal homeostasis and mucosal healing, suggesting it as a therapeutic target for inflammatory bowel disease.

## Abstract 2

miR-147 protects against intestinal inflammation by regulating immune responses and mucosal healing. Its absence impairs healing, suggesting miR-147 as a potential target for inflammatory bowel disease treatment.

## Introduction

The intestinal epithelium supports the absorption of nutrients and water, and provides a physical barrier separating the lumen from the lamina propria (LP) of the intestine. Intestinal epithelium is intelligent and adaptable as it rejuvenates itself, retains integrity, and responds to the presence of pathogenic microorganisms. Disruption of this homeostasis leads to detrimental consequences, which allow intestinal microflora to come in contact with the LP and lead to the initiation of immune responses. A prolonged challenge to intestinal homeostasis is thought to underlie chronic intestinal diseases such as Crohn disease (CD) and ulcerative colitis (UC). Intestinal epithelial cells (IECs), descended from stem cells at the bottom of intestinal crypts, divide and differentiate as they move toward the luminal surfaces where cells adopt their postmitotic fate. This process is essential for repairing and restoring homeostatic function of the mucosal barrier and is compromised during chronic bowel inflammation. A delay or impairment in epithelial wound healing promotes chronic inflammation, contributing to progression of inflammatory bowel disease (IBD) and increasing the risk of colorectal cancer (1). MicroRNAs (miRNAs) are small (21–25 nucleotides) noncoding RNAs regulating gene expression by interacting with the 3′-UTR of their target mRNA and inducing degradation of the transcript (2). Physiological growth and development of eukaryotes require miRNA activity, and aberrant expression of miRNAs is associated with human diseases (3, 4). Intestinal homeostasis is regulated differently in comparison with other sites in the body, as constant exposure to the microbes requires specific regulatory mechanisms to prevent runaway inflammatory responses and intestinal miRNAs can function as rheostats that buffer such signals. Examples of miRNAs in IECs include miR-146a, which reduces inflammatory response in ischemia/reperfusion injury (5), miR-124 that increases protection against pediatric UC (6), miR-375 that mediates the mucosal-immune system crosstalk necessary for protective Th2 responses in parasitic infections (7), and miR-31 that promotes epithelial regeneration in IBD (8). Moreover, our recent study identified hypoxia-driven miR-29a in a regulatory feedback loop that serves to dampen Th1 T cell–mediated intestinal inflammation by limiting activation of pathogenic lymphocytes (9). The specific functions of intestinal miRNAs are of particular interest as these small, portable molecules hold potential as specific therapeutic agents in patients suffering from IBD (10).

miR147 is a highly conserved miRNA derived from a common transcript of the *AA467197/miR147* (normal mucosa of esophagus-specific gene 1, NMES1, C15ORF48) gene (11), initially identified in mouse splenic tissue (12). Murine macrophages upregulate miR147 upon stimulation with Toll-like receptor ligands and lung cells induce miR147 upon LPS treatment, implying a negative feedback loop that diminishes the proinflammatory responses (13). Subsequent studies showed that miR147 participates in various biological processes that include proliferation, migration, and cell death (13–15), making this miRNA a potential cancer biomarker and a therapeutic target in inflammatory diseases. Previous studies have addressed the intestinal role of *AA467197/miR147* deletion (16); however, the specific functional role of miR147 in intestinal inflammation is unknown.

Here, we report that miR147 exhibits its highest expression in the digestive tract, particularly in the colon, as demonstrated using a *miR147tdTomato* reporter mouse. We further identified differentiated IECs as the source of the highest expression of miR147 in the gut. Finally, we mapped the expression of this miRNA to specific absorptive cell populations, enterocytes/colonocytes, within the mouse and human epithelial cells of the large intestine. The homoeostatic expression pattern of miR147 could be recapitulated by growing intestinal organoids in vitro, and furthermore, miR147 expression was enhanced in the colonocytes of *miR147tdTomato* reporter animals during dextran sodium sulfate–induced (DSS-induced) colitis. Importantly, we found that global or IEC-specific deletion of *miR147* dramatically enhanced colitis pathology in DSS-induced colitis. These findings suggest an important role for miR147 in intestinal homeostasis during inflammation, where it aids in the maintenance of tissue integrity and the differentiation process of luminal IECs.

## Results

### High expression of miR-147 in the gastrointestinal tract.

**Figure 1:**
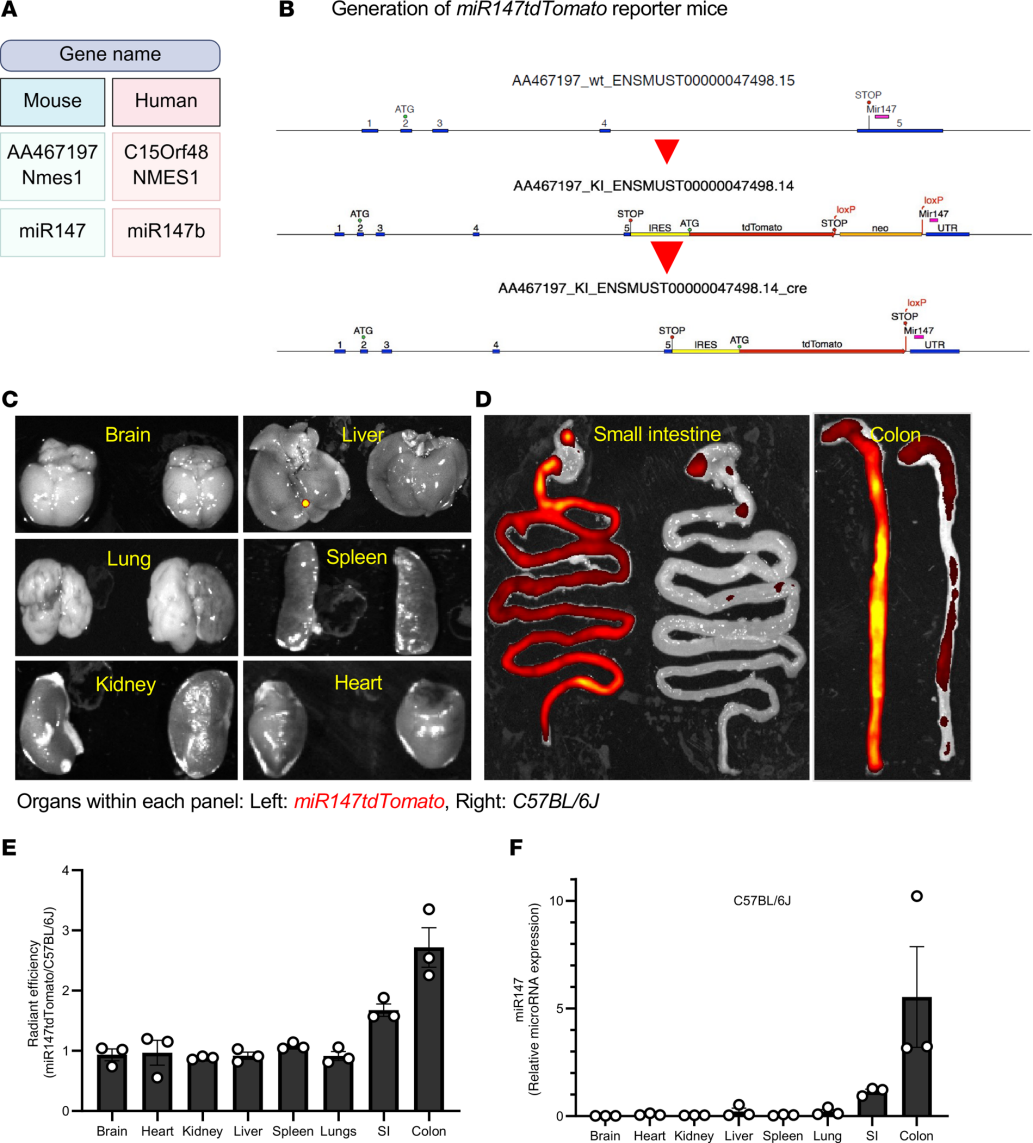
miR147 is highly expressed in the gastrointestinal tract. Expression of *miR147* was examined using a *miR147tdTomato* reporter mouse. (**A**) Nomenclature for mouse and human genes within the *miR147* locus used throughout the text and figures is shown (created with BioRender). (**B**) Schematic of the reporter allele in which the *tdTomato* gene, preceded by an internal ribosomal entry site (IRES), was inserted immediately after the stop codon of *AA467197* and directly before the *miR147* sequence, enabling polycistronic expression from a common promoter. (**C**) Representative IVIS images of dissected organs (brain, lung, kidney, liver, spleen, heart) from a *miR147tdTomato* reporter mouse and a WT (C57BL/6J) control. (**D**) Fluorescent signal detected in the small intestine (left) and colon (right) of a *miR147tdTomato* mouse. (**E**) Quantification of relative fluorescent signal from each organ of *miR147tdTomato* mice, normalized to WT controls (*n* = 3 mice per group). (**F**) qPCR analysis of *miR147* expression in the indicated organs from C57BL/6J mice, normalized to U6 snRNA. Data in **D** and **E** are presented as mean ± SEM; *n* = 3 mice per group per experiment, both male and female mice were used for analysis.

The miR147 locus consists of the host gene *AA467197* (*Nmes1*) and *microRNA-147*, located in the 5th exon of the host gene within the 3′-UTR; this arrangement is highly conserved in vertebrates. In the human genome, these correspond to *C15ORF48* (*NMES1*) and *miR147b* (Figure 1A). To characterize miR147 expression, we generated a miR147 reporter mouse by inserting an *IRES-tdTomato* sequence immediately after the *AA467197* stop codon (Figure 1B). Mice with *miR147tdTomato* exhibited no overt phenotype and were indistinguishable from C57BL/6J (WT) controls. We harvested organs from reporter animals for IVIS imaging. At baseline, brain, heart, kidney, liver, spleen, and lung showed no differential fluorescence versus WT organs (Figure 1C). In contrast, gastrointestinal tissues, including stomach and small intestine, showed appreciably higher fluorescence in *miR147tdTomato* mice (Figure 1D), with the highest signal in colon and rectum (Figure 1D). We note that the alimentary system produces increased autofluorescence detectable by IVIS, as seen in controls, likely from residual luminal contents after PBS flushing (Figure 1D). Fluorescence for each organ group was normalized to controls, and averages are shown in Figure 1E. Quantitative real-time PCR (qPCR) analysis of miR147 in WT mice revealed an expression pattern closely matching that of the reporter (Figure 1F), validating our reporter model for further study.

We conclude that in unchallenged mice, miR147 is expressed highest in the gastrointestinal system, particularly the small intestine and colon.

### Luminal IECs express high levels of miR147.

**Figure 2:**
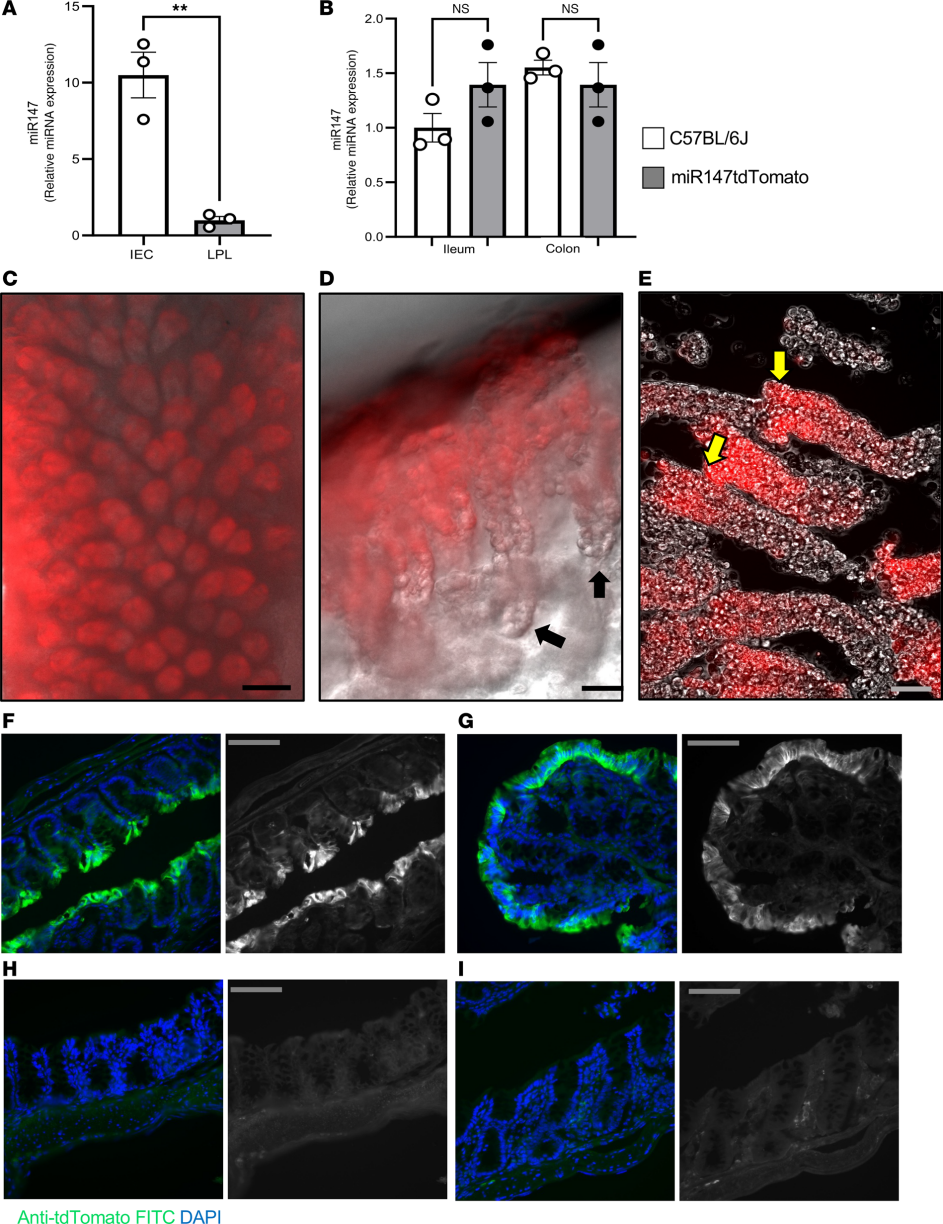
Luminal intestinal epithelial cells express high levels of miR147. (**A**) Colons from C57BL/6J mice were used to purify intestinal epithelial cells (IECs) and lamina propria lymphocytes (LPLs), and expression of miR147 was analyzed by qPCR. (**B**) Comparison of miR147 expression levels in ileal and colonic IECs derived from *miR147tdTomato* and C57BL/6J mice by qPCR. *miR147tdTomato* reporter mice were used to map miR147 expression within the large intestine. (**C**) Representative image of the luminal surface of the colon showing fluorescent *tdTomato* expression. (**D**) Portion of the lamina propria with remaining colonic crypts showing miR147 expression localized to the upper (luminal) portion of the crypts. Black arrows indicate the stem cell areas that lack reporter expression. (**E**) Purified intact colonic crypts visualized under differential interference contrast (DIC) and fluorescent light; yellow arrows indicate the luminal portion of the crypts with visible *tdTomato* expression. (**F**–**I**) Validation of *tdTomato* expression in *miR147tdTomato* mice using fluorescent staining. Frozen intestinal sections were stained with primary anti-tdTomato and secondary (Alexa Fluor 488, AF488) and visualized by confocal microscopy. (**F**) Medial colon. (**G**) Proximal colon. Both panels show overlays of *miR147tdTomato* colon stained with anti-tdTomato/secondary and DAPI, with AF488 fluorescence shown in the right panels. (**H**) Overlay of secondary AF488 and DAPI (left) and secondary alone (right). (**I**) C57BL/6J medial colon, showing overlay of anti-tdTomato/secondary and DAPI (left) and anti-tdTomato/secondary alone (right). In **A** and **B**, *n* = 3 mice/group; pooled, expression normalized to U6 snRNA; data presented as mean ± SEM. **P < 0.01 by unpaired *t* test (**A**) or 2-way ANOVA with Šídák’s test (**B**). In **C**–**I**, experiments repeated at least 3 times. Both male and female mice were used. Scale bars: 200 μm (**C**), 50 μm (**D** and **E**), and 100 μm (**F**–**I**).

Having validated our *miR147tdTomato* reporter mouse, we examined which gut cells express miR147. The intestine was fractionated into IECs and LP lymphocytes (LPLs), as shown in Supplemental Figure 2, A and B; supplemental material available online with this article; https://doi.org/10.1172/jci.insight.190466DS1 qPCR of WT colon fractions revealed miR147 localized selectively to IECs, with very low LPL expression (Figure 2A). IEC fractions from the ileum and colon of WT and *miR147tdTomato* mice showed comparable miR147 expression, indicating *tdTomato* insertion does not disrupt normal expression (Figure 2B).

Next, we visualized the mid-colon luminal surface of *miR147tdTomat*o mice under a fluorescence microscope, observing uniform red fluorescence in luminal IECs and crypts (Figure 2C). Incubation with CellStripper (Corning) removed most crypts, revealing miR147-expressing cells in upper colonic crypts, absent in stem and transit-amplifying cells (Figure 2D, black arrows). Isolated crypts confirmed tdTomato expression in lumen-facing cells (Figure 2E, yellow arrows).

To validate reporter expression, we stained frozen colon sections from *miR147tdTomato* mice with an anti-tdTomato antibody and Alexa Fluor 488–conjugated secondary antibody, with DAPI for nuclei. Middle and proximal colon sections showed tdTomato at luminal surfaces (Figure 2, F and H), absent in WT controls (Figure 2G) or secondary-only staining (Figure 2I). 3D confocal projections (Supplemental Figure 1, A–C, and Supplemental Videos 1–3) confirmed strong tdTomato expression in differentiated luminal cells, establishing miR147 as a marker of differentiated IECs, absent in undifferentiated cells.

### Intrinsic and extrinsic signals drive miR147 expression.

**Figure 3:**
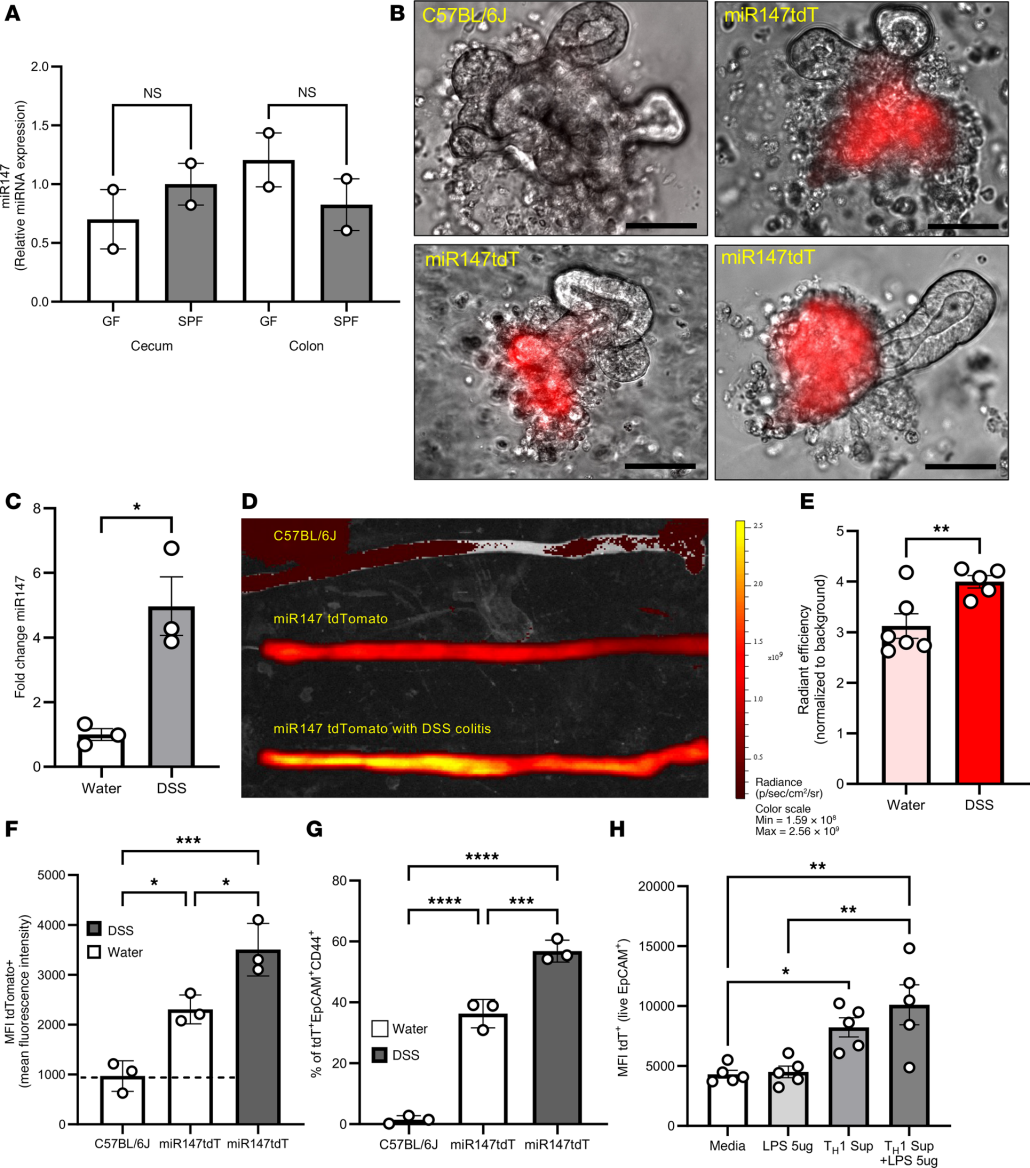
Intrinsic and extrinsic signals drive miR147 expression. Colons and ceca from germ-free (GF) male mice and age- and sex-matched specific pathogen–free (SPF) C57BL/6J mice were used to isolate intestinal epithelial cells (IECs). (**A**) Expression of miR147 in GF- and SPF-derived IECs was analyzed by qPCR (*n* = 2 mice per group). (**B**) *miR147tdTomato* and C57BL/6J ceca were used to derive organoid cultures. Individual live organoids on day 13 (passaged once) were imaged under DIC and fluorescent light; representative overlaid images are shown. Repeated twice, *n* = 2–3 mice per group. (**C**) Expression of miR147 during inflammation was studied using a DSS colitis model in which mice received 3% DSS for 5 days followed by 2 days of water. Colonic RNA was purified and analyzed for miR147 expression by qPCR (*n* = 3 mice per group). Scale bars: 50 μm. (**D**) Representative IVIS image of colon fluorescence in *miR147tdTomato* DSS-treated mice and controls. (**E**) IVIS signal from colons was quantified and expressed as relative efficiency, normalized to C57BL/6J background fluorescence. Two pooled experiments, 2–3 mice per group. (**F**) Total IECs from *miR147tdTomato* and control mice treated with 3% DSS were isolated, stained for live cells, and analyzed for tdTomato fluorescence by flow cytometry. (**G**) IECs as in **F**, gated on live/EpCAM^+^ and CD44^+^, were analyzed for tdTomato expression by flow cytometry. One of 2 experiments is shown (*n* = 3 mice per group). (**H**) Isolated IECs were cultured for 18 hours with indicated stimuli, gated on live/EpCAM^+^, and analyzed by flow cytometry; 2 pooled experiments are shown. Data are presented as mean ± SEM and were analyzed by 2-tailed *t* test (**C** and **E**) or 1-way ANOVA with Šídák’s test (**A**), Tukey’s 2-tailed *t* test (**F** and **G**), or Holm-Šidák test (**H**). *P < 0.05; **P < 0.01; ***P < 0.001; ****P < 0.0001. In **B**–**H**, both male and female mice were used.

Intrigued by the very high miR147 expression in alimentary epithelia, we investigated its regulation. The intestinal system hosts abundant microflora, with the highest concentration in the colon. Since miR147 expression mirrors microbial concentration in the gut, we hypothesized that germ-free mice would show diminished miR147 in intestinal epithelium. However, miR147 levels were comparable between cecal and colonic epithelial cells from germ-free and age/sex-matched specific pathogen–free mice (Figure 3A), suggesting expression is not driven by the presence of microflora. Intestinal organoids recapitulate many aspects of colon structure and function. We isolated colonic and cecal crypts from *miR147tdTomato* and control mice and cultured them in Matrigel with growth factors. Freshly isolated crypts showed strong reporter expression, which largely disappeared by day 3 as mature intestinal cells died (Supplemental Figure 3A). Newly growing organoids expressed little to no tdTomato in proliferating ends but accumulated red fluorescence in mature cells in older regions (Figure 3B and Supplemental Figure 3A, day 7). These data indicate baseline miR147 expression is driven by intrinsic epithelial cell differentiation rather than extrinsic factors like luminal microorganisms.

Next, we studied the effect of intestinal inflammation on miR147 expression, as dysregulation was previously noted in patients with colon cancer (17, 18). WT mice treated with 3% DSS showed increased miR147 expression in colonic tissue (Figure 3C). Similarly, *miR147tdTomato* reporter mice exhibited increased fluorescence in the colon after DSS treatment (Figure 3D), with a statistically significant difference between treated and untreated cohorts (Figure 3E and Supplemental Figure 3B). Flow cytometry of isolated colonic epithelial cells revealed increased fluorescence per cell (Figure 3F). Gating on EpCAM^+^ and CD44^+^ cells (markers of committed IECs; Supplemental Figure 3C) confirmed DSS-induced miR147 upregulation (Figure 3G), while IEC survival was unaffected (Supplemental Figure 3D). To examine inflammatory signals inducing miR147, purified IECs were treated in vitro with LPS, inflammatory conditioned media (supernatant from Th1 T cells), or both. Consistent with previous findings (19), tdTomato expression increased significantly with conditioned media alone or combined with LPS, but not with LPS alone (Figure 3H). qPCR of RNA from similarly treated WT IECs confirmed increased miR147 expression with conditioned media alone or with LPS (Supplemental Figure 3E). These results indicate baseline miR147 expression is cell intrinsic, while inflammation induces further upregulation in vivo and can be modeled in vitro by proinflammatory conditioning.

### Epithelial miR147 protects intestinal integrity during inflammation.

**Figure 4:**
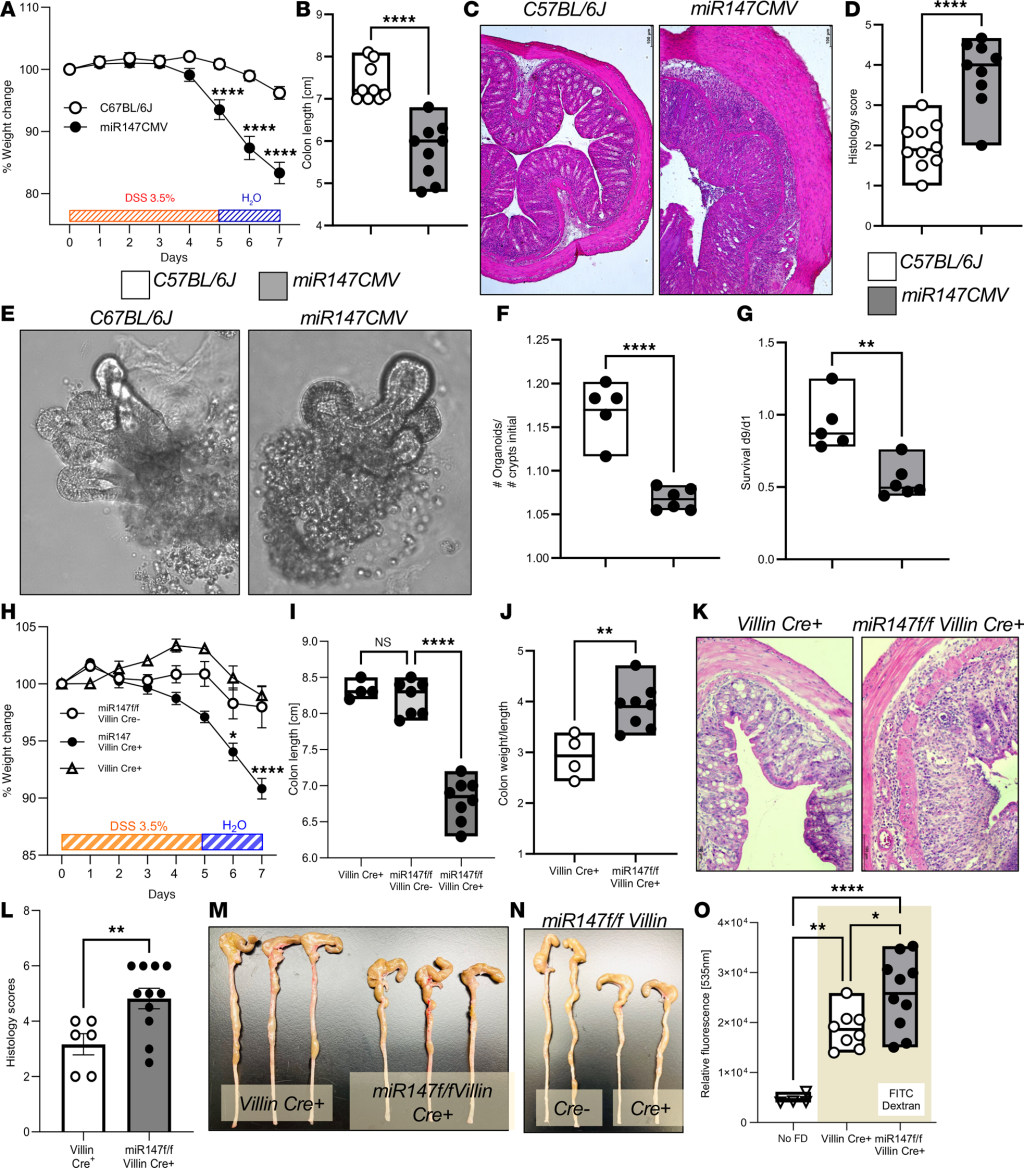
Epithelial miR147 protects intestinal integrity during inflammation. Mice deficient in miR147 (*miR147CMV*) and controls (C57BL/6J) were treated with 3.5% DSS in drinking water followed by 2 days of water. (**A**) Weight change in the experimental groups. (**B**) Colon length measured at the conclusion of the experiment. (**C**) Representative medial colon histology from control and miR147-deficient mice. (**D**) Histopathology scoring (lymphocyte infiltration and epithelial damage) in miR147-deficient mice and controls. Organoids from *miR147CMV* and C57BL/6J mice were cultured with growth monitored daily. (**E**) Representative organoid images on day 9. (**F**) Organoid initiation rates per plated crypt over 9 days. (**G**) Organoid survival on day 9. Data in **A**–**D** represent pooled results from 2 experiments (9–10 mice/group). Data in **E**–**G** represent 2 mice per group. Male mice were used. Mice with intestinal epithelium–specific miR147 deficiency (*miR147^fl/fl^* *Villin Cre^+^*) and controls (*Villin Cre^+^* and *miR147^fl/fl^* *Villin* *Cre^–^*) were treated with 3.5% DSS followed by 2 days of water. (**H**) Weight change during the experimental period. (**I**) Colon length at the end of the experiment. (**J**) Colon weight/length ratio. (**K**) Representative micrographs of the colons. (**L**) Inflammation score from colon histological sections. Representative colon length images in DSS-treated mice are shown for *miR147^fl/fl^* *Villin Cre^+^* and *Villin Cre^+^* (**M**), or *miR147^fl/fl^* *Villin Cre^+^* and littermates (**N**) on day 4. (**O**) On day 5 of DSS treatment, mice were fasted, orally gavaged with FITC-dextran, and intestinal permeability was measured by serum FITC-dextran detection. Data shown as mean ± SEM; pooled results from 2 experiments; *n* = 8–11 male mice/group. *P < 0.05; **P < 0.01; ****P < 0.0001 by 1-way ANOVA (**A** and **I**), 1-way ANOVA with Šidák’s test (**H**) or Tukey’s test (**G** and **O**), or unpaired 2-tailed *t* test (**B**, **D**, **F**, **J**, and **L**).

Encouraged by evidence that inflammation drives miR147 expression, we tested the effect of miR147 deficiency in a colitis model. Mice with whole-body *miR147* deletion (*miR147CMV*, Cre allele lost) and WT controls were treated with 3.5% DSS in drinking water, followed by 2 days of water washout; tissues were harvested on day 7. miR147-deficient mice exhibited greater body weight loss during DSS treatment (Figure 4A). Their colons were significantly shorter than WT controls (Figure 4B). Histological analysis revealed increased epithelial damage in miR147-deficient colons, as shown in representative H&E-stained micrographs (Figure 4C). Blinded scoring confirmed a significantly higher inflammatory index in miR147-deficient colonic tissues (Figure 4D).

We hypothesized that increased epithelial damage in miR147-deficient animals may reflect epithelial deficiency. To test this, we prepared intestinal organoids and tracked their growth for 9 days. Organoids that successfully initiated growth showed similar overall morphology between groups (Figure 4E). However, *miR147CMV*-derived organoids had lower initiation rates (Figure 4F) and diminished survival (Figure 4G) compared with WT. Whole-body miR147 deletion leads to severe DSS colitis outcomes, with organoids from miR147-deficient mice compromised in initiation and growth.

While whole-body *miR147* deletion causes severe weight loss and intestinal damage in DSS colitis, many cell types contribute to this pathology. To specifically test miR147’s role in intestinal epithelia, we derived *miR147^fl/fl^* *Villin Cre* mice and assessed their DSS response. After 3.5% DSS for 5 days plus 2 days of water, epithelial miR147 deletion caused significant weight loss versus controls (Figure 4H). Colons from *miR147^fl/fl^* *Villin Cre^+^* mice were shorter than *Villin Cre^+^* or *miR147^fl/fl^* *Villin Cre^–^* littermates (Figure 4I), with higher weight-to-length ratios indicating increased inflammation (Figure 4J). H&E-stained medial colon micrographs showed increased epithelial damage and leukocyte infiltration in epithelial mutants (Figure 4K), with elevated histological inflammation scores (Figure 4L). Shortened colons on DSS day 4 are shown in Figure 4M. Comparing *miR147^fl/fl^* *Villin Cre^+^* and *Cre^–^* littermates confirmed colon shortening differences were due to epithelial miR147 loss, not microflora variations (Figure 4N). Fecal microbiome analysis (20) found no differences in relative abundance or composition between co-housed *miR147^fl/fl^* *Villin Cre^+^* and *Cre^–^* littermate mice (Supplemental Figure 4).

Mice with compromised intestinal epithelial homeostasis may show gut barrier leakiness during inflammation (21). To test whether miR147 contributes to barrier integrity, we gavaged mice with FITC-dextran on day 5 of DSS treatment and measured serum fluorescence after 4 hours. *miR147^fl/fl^* *Villin Cre*^+^ mice showed increased intestinal permeability compared with controls (Figure 4O). Together, these results demonstrate that deletion of miR147 in the intestinal epithelium recapitulates the phenotype of a whole-body miR147 deletion in the DSS colitis model. miR147 plays a crucial role in the maintenance of intestinal integrity and its deletion in the intestinal epithelium leads to a pronounced inflammation-induced damage, leakiness of the gut barrier layer, and a severe colitis phenotype.

### Deletion of miR147 disrupts immune signaling pathways in the intestine.

**Figure 5:**
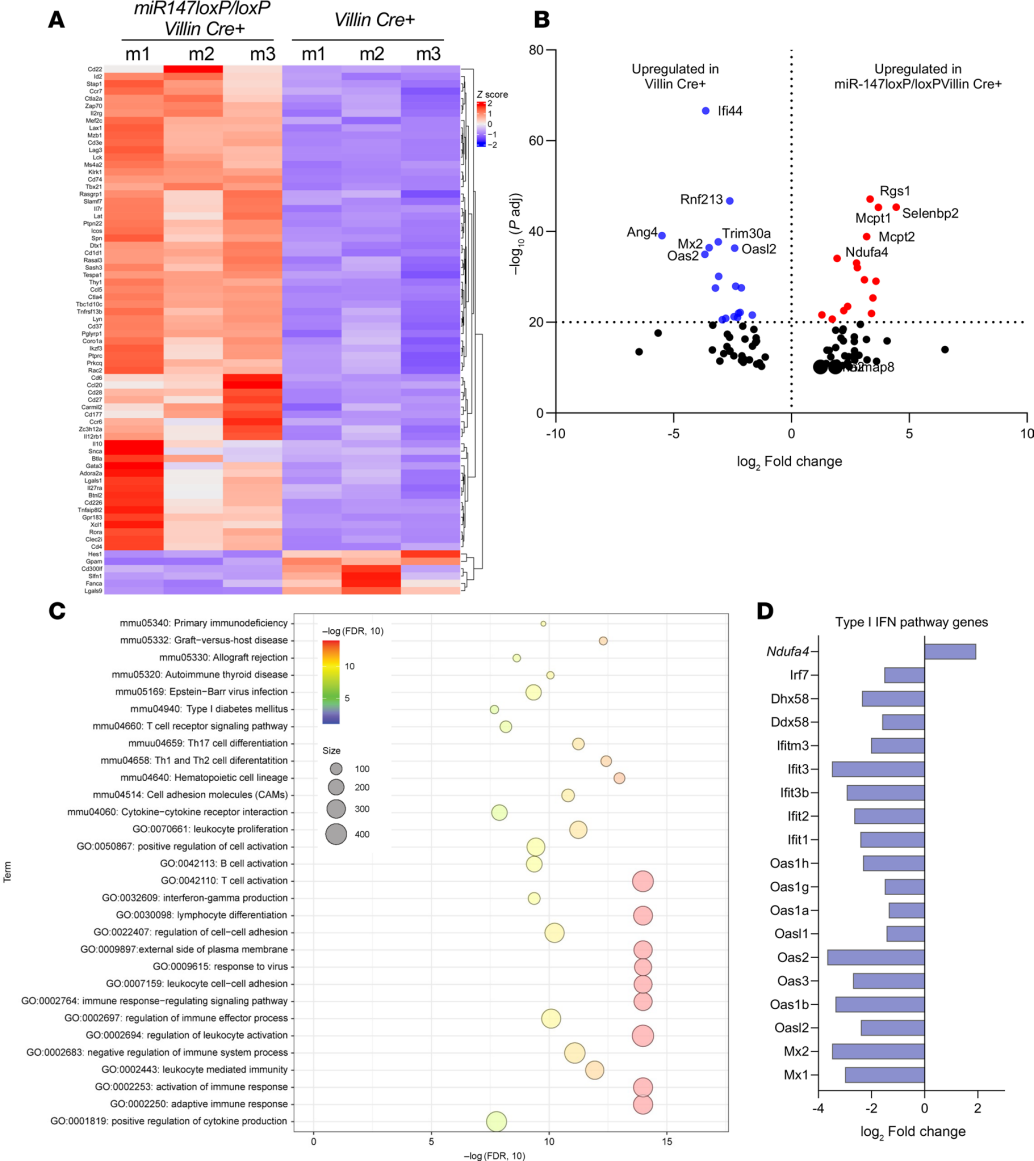
Deletion of miR147 disrupts immune signaling pathways in the intestine. Total RNA was purified from isolated intestinal epithelial cells (IECs) derived from *iR147^fl/fl^* *Villin Cre^+^* and *Villin Cre^+^* mice (*n* = 3 per group) treated with 3% DSS for 5 days followed by 1 day of water. RNA-seq was performed and analyzed at the UT Health Science Center Cancer Genomic Core. (**A**) Heatmap of downregulated and upregulated genes across each group of 3 mice. (**B**) Volcano plot of the top 100 differentially expressed genes (DEGs), based on adjusted *P* value and log_2_(fold change), between the 2 groups. Red dots represent genes expressed at higher levels in *miR147^fl/fl^* *Villin Cre^+^* mice; blue dots represent genes with higher expression levels in *Villin Cre^+^* mice. The *x* axis represents log_2_(fold change); the *y* axis represents statistical significance (–log_10_ *P*_adj_) for each gene, analyzed by DESeq2. The volcano plot was generated using GraphPad Prism version 10.1.1. (**C**) Gene ontology (GO) enrichment analysis of DEGs retrieved using DAVID (https://david.ncifcrf.gov/). The top 30 most enriched GO terms in the biological process, molecular function, and cellular component categories are presented. All adjusted statistically significant values of the terms were –10-base log transformed. (**D**) Bar graph showing the top genes, based on log_2_(fold change), related to the type I interferon pathway in the miR147-deficient intestinal epithelium, analyzed by DESeq2.

To understand miR147’s role in the intestinal epithelium, we assessed gene expression changes after miR147 deletion in IECs. Matched cohorts of *miR147^fl/fl^* *Villin Cre^+^* and *Villin Cre^+^* mice were treated with 3% DSS for 5 days, followed by 1 day of water, and then colonic epithelial cells were harvested for total RNA. RNA sequencing (RNA-seq) revealed differential gene expression profiles (Figure 5A). Of note, in addition to a majority of upregulated genes, a marked number of genes was also downregulated in miR147-deleted epithelial cells (see below). Among the top 5 upregulated genes was the mitochondrial complex IV–associated gene Ndufa4 (Figure 5B). Prior studies identified Ndufa4 as a miR147 target in other cell types (11, 22, 23), but its targeting in IECs was not studied. Ndufa4 is a mitochondrial inner membrane protein interacting with complex IV of the electron transport chain. Numerous immune pathways were dysregulated in miR147-deficient IECs, including cell adhesion and cytokine-receptor signaling (Figure 5C). Among downregulated genes, many type I interferon response genes stood out (Figure 5D). These RNA-seq results in miR147-deficient IECs under inflammatory conditions identify Ndufa4 as a key target and reveal dysregulation of multiple immune signaling pathways in the intestinal epithelium.

### Ndufa4 is a target of miR147 in the intestinal epithelium.

**Figure 6:**
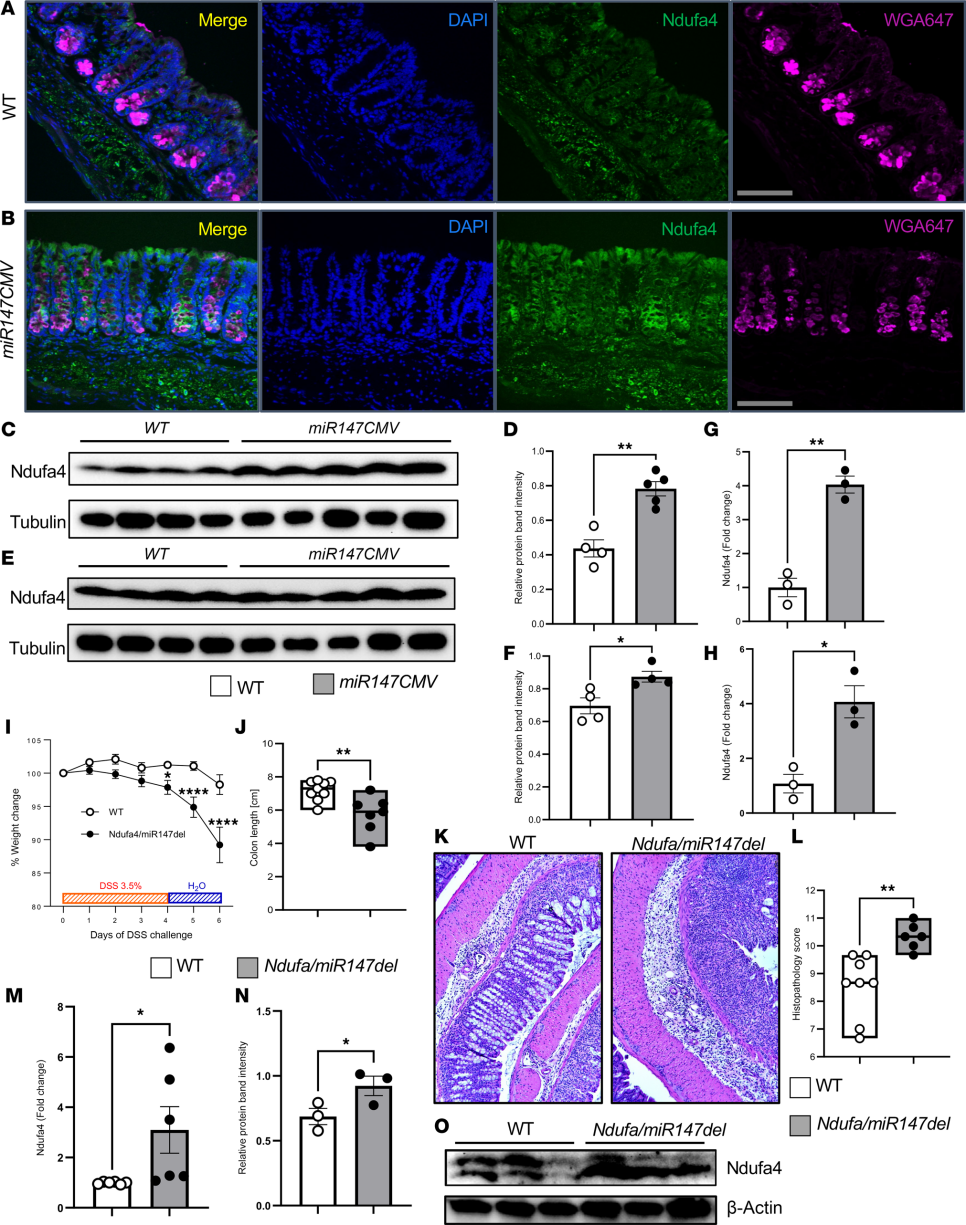
Ndufa4 is a target of miR147 in intestinal epithelium. *miR147CMV* and WT mice were treated with 3% DSS for 3 days (*n* = 3, repeated twice). (**A**) Frozen sections from WT mice stained with anti-Ndufa4 antibody. Nuclei were stained with DAPI, mucus granules with WGA. (**B**) Similarly stained sections from *miR147CMV* mice. Protein extracts from 3% DSS–treated mice were resolved on acrylamide gels (*n* = 4–5). Scale bars: 100 µm. (**C**) Ndufa4 expression in cecum. (**D**) Relative abundance of Ndufa4 bands quantified by ImageJ. (**E**) Colonic Ndufa4 expression. (**F**) Relative abundance of Ndufa4 bands quantified by ImageJ. RNA from IECs of miR147CMV and WT mice was analyzed by qPCR. (**G**) miR147 expression in colonic IECs. (**H**) RNA from colonic IECs of *miR147CMV* and WT mice treated with 3% DSS for 3 days followed by 1 day of water was analyzed for Ndufa4 expression by qPCR (*n* = 3–5). Mice lacking the miR147 binding site in the Ndufa4 3′-UTR (*Ndufa4/miR147del*) and WT (*CMV Cre*) were treated with 3.5% DSS followed by 2 days of water. (**I**) Weight change in experimental groups. (**J**) Colon length at the end of the experiment. (**K**) Representative medial colon histology in control and miR147-deficient mice. (**L**) Histopathology evaluation in miR147-deficient mice and controls. (**M**) RNA from IECs of *Ndufa4/miR147del* and WT mice analyzed for Ndufa4 expression by qPCR (*n* = 6). (**N**) Relative abundance of Ndufa4 in IECs, quantified by ImageJ. (**O**) Ndufa4 expression in IECs from WT and *Ndufa4/miR147del* mice. Pooled data from 2 experiments (*n* = 7–8 in **I**–**K**). Data are presented as mean ± SEM. *P < 0.05; **P < 0.01; ****P < 0.0001 by unpaired 2-tailed *t* test (**D**, **F**, **G**, **H**, **J**, and **L**), 1-way ANOVA with Šídák’s test (**I**), or 1-tailed *t* test (**M** and **N**). Male mice used throughout.

Encouraged by RNA-seq findings suggesting miR147 targets Ndufa4 in IECs, we validated this by assessing Ndufa4 expression in the epithelium. Ndufa4-specific antibody was used to stain frozen sections of miR147-deficient and control intestines. As shown in Figure 6A, Ndufa4 was detected in all intestinal epithelial compartments, including the LP and crypt epithelial cells. Expression appeared more pronounced and brighter in miR147-deficient intestines, specifically in differentiated epithelial cells where WT expression was lower (Figure 6B, Supplemental Figure 5, A and B, and Supplemental Videos 4 and 5). Fluorescent wheat germ agglutinin (WGA) labeling showed similar goblet cell mucosal granule numbers in both strains. Total protein extracts from cecal and colonic epithelial cells showed stronger Ndufa4 bands in miR147-deficient mice (Figure 6C), with ImageJ quantitation confirming a marked increase (Figure 6D). Colonic epithelial cells also showed higher Ndufa4 expression in miR147-deficient mice (Figures 6, E and F). RNA from IECs confirmed miR147 absence in knockouts (Supplemental Figure 5C) and significantly higher Ndufa4 levels in colonic (Figure 6G) and small intestinal epithelium (Supplemental Figure 5D). AA467197 (Nmes1) expression remained comparable between genotypes (Supplemental Figure 5E). These results contrast with models deleting both Nmes1 and miR147 (16). Finally, IEC RNA from mice treated with DSS for 5 days showed increased Ndufa4 in miR147-deficient mice versus controls (Figure 6H). These results demonstrate miR147 suppresses Ndufa4 expression in IECs, with miR147 deficiency causing robust baseline and DSS-induced Ndufa4 protein and mRNA upregulation.

To address miR147-mediated targeting of Ndufa4 at the transcript level, we used the transgenic *Ndufa/miR147del* mouse, where the putative miR147 binding site in the 3′ region of *Ndufa4* gene was deleted. When challenged with 3.5% DSS, *Ndufa/miR147del* mice exhibited increased disease severity, shown by marked weight loss (Figure 6I), shortened colon (Figure 6J), and dramatically increased tissue damage (Figure 6K). Histological inflammation scoring is shown in Figure 6L. Epithelial cells from *Ndufa/miR147del* colons had increased *Ndufa4* mRNA (Figure 6M) and elevated Ndufa4 protein expression (Figures 6, N–O). Thus, *Ndufa/miR147del* mice phenocopy *miR147^fl/fl^* *Villin Cre^+^* mice in colitis, demonstrating miR147’s role in suppressing Ndufa4 expression in the intestinal epithelium. These findings indicate Ndufa4 as a functionally regulated target of miR147 in IECs, likely through its 3′-UTR, and underscore the critical role of this regulatory axis in maintaining mucosal homeostasis during colitis.

### AA467197/miR147 and Ndufa4 define distinct epithelial cell populations in the intestine.

To investigate spatial expression of miR147 and Ndufa4 across colonic epithelium and cell types, we performed spatial transcriptomics using Visium HD on colonic Swiss roll sections from a healthy WT mouse (n981_healthy; 386,865 pseudo-cells) and a DSS-treated WT mouse (n2_DSS; 421,574 pseudo-cells) modeling inflammation. Data quality was assessed by standard metrics, including gene counts and mitochondrial content, with consistent filtering thresholds applied to both samples to ensure high-confidence gene expression profiles (Supplemental Figures 6 and 7).

**Figure 7:**
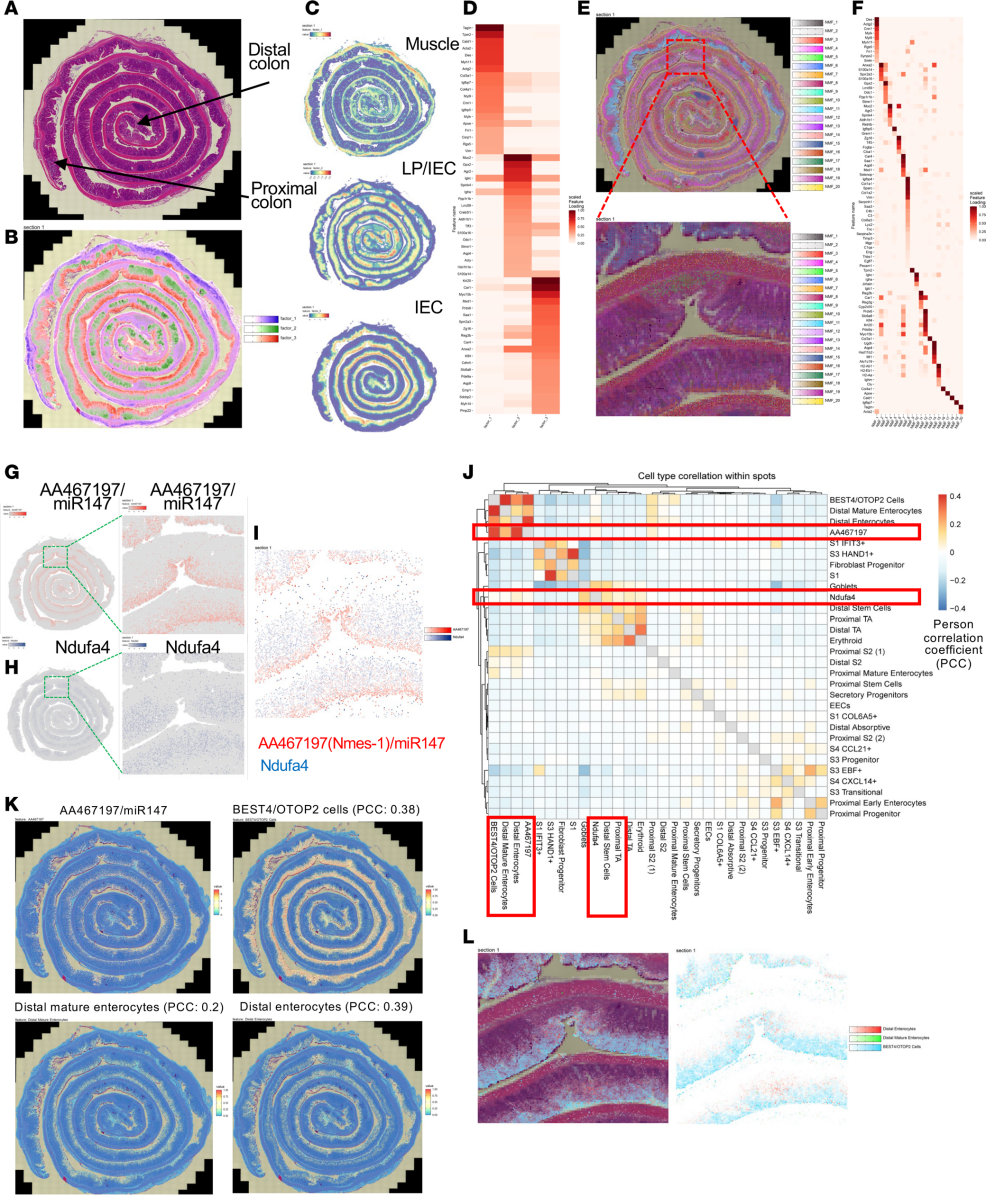
AA467197/miR147 and Ndufa4 define distinct epithelial cell populations in the intestine. Three factors identify the primary tissue structures of the murine colon from the n981_healthy sample. (**A**) Colonic Swiss rolls shown with H&E staining. (**B**) Spatial transcriptomics (ST) spots color-coded based on non-negative matrix factorization (NMF). ST spots uniquely assigned to 1 factor are colored red, blue, and green for factor 1, 2, and 3, respectively. ST spots shared between factors are colored with intermediate gradations of these 3 colors. (**C**) Spatial distribution of the 3 factors distinguishing muscle, lamina propria, and intestinal epithelial cells. (**D**) Heatmap showing the top 20 genes defining each factor. Spatial distribution of 20 factors in the murine colon of the n981_healthy sample. (**E**) Colonic Swiss rolls with ST spots color-coded based on 20 NMF factors, each assigned a color-coded score reflecting gene expression defining each factor. (**F**) Heatmap showing the top genes defining each factor. (**G**) AA467197/miR147 and (**H**) Ndufa4 transcript expression in colonic Swiss rolls of the n981_healthy sample. (**I**) Superimposed expression patterns of AA467197/miR147 and Ndufa4 in a selected tissue area. Human cell type mapping onto the murine spatial transcriptomic dataset. (**J**) Pearson correlation matrix between human single-cell transcriptomic signatures and mouse ST spot-level expression of AA467197/miR147 and Ndufa4 in the n981_healthy sample. (**K**) Spatial feature plots on murine colonic Swiss roll sections showing AA467197/miR147 expression (upper left) alongside inferred cell-type proportions for key populations, as calculated by NNLS deconvolution. (**L**) Spatial plots on murine colonic Swiss roll sections displaying inferred cell-type proportions for key populations.

To further elucidate the transcriptional landscape influenced by miR147 and Ndufa4, we normalized the gene expression matrix using NormalizeData() in Seurat, and then applied non-negative matrix factorization (NMF) with RunNMF(), constraining the model to 3 factors to delineate primary colonic structures: LP, IECs, and muscle. Examination of the highest-loading genes supported their assignment to muscle, IECs, and a mixed profile of IECs, muscle, and LP. Spatial localization of these factors, corresponding to muscle, LP, and epithelial compartments, is shown in Figure 7C, with a heatmap of the top 20 genes per factor in Figure 7D.

To examine gene expression variation across colon regions, dimensionality reduction and clustering identified 20 transcriptional clusters representing unique cell populations or tissue regions. Spatial patterns were visualized by assigning each spatial transcriptomics spot a color-coded score based on genes from 20 transcriptional programs derived by NMF (Figure 7E). A heatmap of top differentially expressed genes defining each cluster is shown in Figure 7F, with spatial mapping correlating clusters to anatomically distinct colon regions (Supplemental Figure 8). These findings demonstrate conserved regional organization of gene expression in the healthy colon.

To further investigate the spatial localization of the miR147 transcript (AA467197/Nmes1) and its predicted target Ndufa4, we projected their expression intensities across the tissue using spatial mapping functions (MapFeatures and MapMultipleFeatures). In the healthy colon sample (n981_healthy), miR147 expression was highly localized to defined epithelial regions (Figure 7G), while Ndufa4 showed a complementary distribution, enriched in distinct zones of the tissue (Figure 7H). Overlaying both signals revealed mutually exclusive expression domains, supporting the notion of cell-type-specific regulation within the colonic epithelium (Figure 7I). This spatial separation suggests that miR147 and Ndufa4 occupy distinct functional niches in the colon, suggesting differing roles during epithelial differentiation. To further investigate how epithelial cell states and transcriptional programs are altered during inflammation, we performed joint embedding of pseudo-cell transcriptomes from healthy (n981_healthy) and DSS-treated (n2_DSS) colonic tissues. Uniform manifold approximation and projection (UMAP) integration revealed a shared transcriptional landscape, with cells from both conditions overlapping across clusters, indicating preservation of major epithelial and stromal cell types (Supplemental Figure 9A). Subsequent graph-based clustering identified 23 distinct transcriptional states (Supplemental Figure 9B), providing a unified reference map for comparing spatial gene expression, AA467197/miR147 and Ndufa4 localization, and deconvolved human cell-type proportions across homeostasis and inflammation.

To link gene expression with specific epithelial cell types, we applied non-negative least squares (NNLS) deconvolution to the murine spatial transcriptomic data using a human single-cell reference atlas of gut epithelial and mesenchymal populations (24). After intersecting the top 10,000 variable genes and balancing cell-type representation, we estimated the contribution of each cell type across spatial spots. The resulting maps (Figure 7J) revealed distinct spatial associations between gene expression and epithelial lineages. miR147 (AA467197/Nmes1) expression correlated strongly with BEST4^+^OTOP2^+^ cells (specialized enterocytes) (Pearson’s correlation coefficient [PCC] = 0.38), distal enterocytes (PCC = 0.39), and distal mature enterocytes (PCC = 0.20), consistent with localization to differentiated, postmitotic epithelial populations. In contrast, Ndufa4 was associated with goblet cells (PCC = 0.14), distal stem cells (PCC = 0.10), and showed notable expression in transit-amplifying cells, suggesting a role in proliferative or metabolically active compartments. Spatial feature plots confirmed these patterns and highlighted the mutually exclusive localization of miR147 (AA467197/Nmes1) and Ndufa4 across the colonic epithelium (Figure 7K). Inferred spatial distributions of the major IEC populations expressing miR147 (AA467197/Nmes1) further illustrates their regional compartmentalization within the tissue (Figure 7L).

We next addressed the expression patterns and cell type specificity of human miR147 (AA467197/Nmes1) homologs, miR147b (C15ORF48/NMES1) and NDUFA4, within the human intestinal epithelium. Using single-cell transcriptomic data from the Pan-GI Cell Atlas (25) (1_Healthy_Pan GI_atlas_all_lineages_20241119.h5ad) and curated level 3 annotations, epithelial subtypes were identified and analyzed via Scanpy (v1.9+). Among epithelial lineages, C15ORF48 expression was most prominent in subsets of differentiated absorptive enterocytes, with colonocytes, mature colonocytes, and BEST4^+^ colonocytes showing the highest levels (Supplemental Figure 10A). In contrast, NDUFA4 was enriched primarily in less differentiated epithelial subsets, especially stem-like, progenitor, and goblet cells. These results align with our murine colon findings, where miR147 (AA467197/Nmes1) and Ndufa4 show a similar mutually exclusive expression pattern across IEC types.

Dot plot analysis revealed both the scaled average expression and proportion of cells expressing C15ORF48 and NDUFA4 across annotated epithelial subtypes. UMAP visualization illustrates the spatial distribution of C15ORF48 expression across epithelial populations, with highest signal intensity in differentiated colonocyte clusters (Supplemental Figure 10B). Conversely, UMAP projection of NDUFA4 showed localization within progenitor-rich and secretory lineages (Supplemental Figure 10C). An integrated UMAP embedding of all IECs confirmed cell-type classification by Louvain clustering and supported subtype-specific expression of both genes (Supplemental Figure 10D). To complement transcriptomic findings, we queried the Human miRNA Tissue Atlas (https://ccb-compute2.cs.uni-saarland.de/mirnatissueatlas_2025) to evaluate organ-level expression of miR147b, a putative posttranscriptional regulator of NDUFA4. Notably, miR147b expression ranked among the highest within the human gastrointestinal tract, supporting its relevance in epithelial lineage function (Supplemental Figure 10E). Integrating the results of our spatial transcriptomics with human single-cell analyses revealed that C15ORF48/miR147b and NDUFA4 exhibited conserved, mutually exclusive expression patterns in the colonic epithelial subsets, highlighting their potential distinct roles in epithelial differentiation and function.

## Discussion

In this study, we identified miR147 as a highly expressed and spatially restricted miRNA in the colon, particularly within the differentiated luminal IECs. Using our *miR147tdTomato* reporter mouse, we showed that miR147 expression increases during epithelial differentiation and is further upregulated by inflammatory signals. Deletion of the isolated *miR147* gene in the whole body or in the intestinal epithelia led to severe disease and a loss of intestinal barrier integrity during DSS-induced experimental colitis. Quantitative gene expression, immunofluorescence, Western blot, and qPCR confirmed that loss of miR147 substantially increases Ndufa4 expression in IECs, both at baseline and during inflammation. We then showed that deletion of the putative miR147 binding site in the Ndufa4 3′-UTR phenocopies deletion of miR147 in the intestinal epithelium, resulting in severe colitis, suggesting miR147 regulates Ndufa4 expression, likely via its 3′-UTR. Moreover, spatial and single cell transcriptomics analyses reveal a conserved, spatially organized expression pattern of AA467197 (Nmes1)/miR147 and Ndufa4 across the colonic epithelium, with miR147 enriched in differentiated absorptive cells and Ndufa4 localized to proliferative and secretory compartments.

Current strategies to combat IBD are almost exclusively aimed at reducing symptoms and increasing patients’ quality of life (26, 27). Recently, a new conceptual approach has emerged where chronic IBD diseases, such as CD and UC, are seen as products of both runaway inflammation and mucosal wounding that does not properly heal. The sustained mucosal damage, which persists even in patients with disease remission, is thought to allow for cyclic disease flare-ups. Conceptually, to cure the disease and stop the flare-up cycles, mucosal wounding and repair must be addressed. Our work, which we believe represents initial steps, focuses on identifying and characterizing genes that regulate mucosal healing and promote intestinal epithelial homeostasis.

Our *miR147tdTomato* reporter mouse, which we believe to be a novel tool, enables in vivo visualization and precise mapping of miR147 expression, facilitating detailed studies of its role in epithelial differentiation and repair. *miR147tdTomato* reporter mice showed a high expression level of miR147, specifically in the differentiated cells of the gut lining. High-definition spatial transcriptomics precisely localized miR147 expression to differentiated colonocytes. These cells highly correlate with diminished Ndufa4 expression, consistent with miR147’s regulatory role. Quantitative gene expression, immunofluorescence, Western blot, and qPCR confirmed that loss of miR147 substantially increases Ndufa4 expression in IECs, both at baseline and during inflammation. We then showed that deletion of the putative miR147 binding site in the Ndufa4 3′-UTR phenocopies deletion of miR147 in the intestinal epithelium, resulting in severe colitis, suggesting miR147 regulates Ndufa4 expression, likely via targeting of its 3′-UTR. While the *Ndufa/miR147del* mouse, RNA-seq, and expression analyses strongly suggest that miR147 regulates Ndufa4 expression, direct biochemical evidence, such as luciferase reporter assays, is needed to confirm miR147’s binding to the Ndufa4 3′-UTR in IECs. Nevertheless, the robust functional evidence from the *Ndufa/miR147del* mouse, which phenocopies miR147-deficient mice in colitis, combined with increased Ndufa4 expression in miR147-deficient IECs and mutually exclusive spatial expression patterns in murine and human colons, strongly supports a regulatory role for miR147 in suppressing Ndufa4 expression to maintain intestinal epithelial homeostasis during inflammation. These findings, supported by spatial transcriptomic mapping, reveal a spatially organized regulatory axis in the colon, where miR147 modulates the metabolic identity of differentiated colonocytes by suppressing Ndufa4. Recent studies link colonocyte energy metabolism to epithelial function and intestinal homeostasis (28, 29) and our study places the AA467197 (Nmes1)/miR147/Ndufa4 axis as an important regulator of enterocyte/colonocyte function during inflammation.

Previous studies indicated a potential role for miR147 in inhibiting metastatic transition in colon cancer cells (15, 30). Another study found an increase in miR147 in the intestines of canines affected with IBD (31). These findings, together with our data reported here, strongly suggest a protective role for miR147 in maintaining intestinal epithelial homeostasis. The small size and portability of miR147 make it a very promising candidate for a mucosal healing agent that could be delivered in the form of a mimetic to promote mucosal repair and potentially halt the progression of intestinal inflammation (32). Unlike Xiong et al. (16), who studied the combined deletion of *AA467197* (*Nmes1*) and *miR147*, our use of a miR147-specific reporter and knockout mouse facilitates attribution of phenotype to miR147 alone, allowing us to define its role in epithelial repair apart from AA467197 (Nmes1). Our miR147-reporter mouse permits us to visualize the expression patterns in vivo by precisely localizing the reporter-positive cells in the colon and ex vivo using intestinal organoids to confirm the in vivo observations. This work, which we believe to be a focused effort, defines the role of miR147 in intestinal epithelial repair and demonstrates that deletion of only miR147 results in increased colitis pathology. Having defined the expression pattern of miR147 specifically to the differentiated epithelium and the enterocytes, our fluorescent reporter mouse model may also provide a valuable tool to visually follow in vivo as well as in vitro differentiation of IECs.

One of the unfortunate consequences of chronic intestinal inflammation is a dramatically increased propensity to develop colon cancer. UC and CD sufferers face worse cancer prognoses than similarly diagnosed IBD-free patients (33, 34). Beyond its role in inflammation, miR147 also appears to influence oncogenic pathways. Expression of miR147 negatively correlates with cancer progression in several tumors, including colorectal adenomas, and forced overexpression of miR147 results in halted tumor cell proliferation (35–37). Conversely, Ndufa4 is associated with cancer-specific survival (38), contributes to the growth and metastasis of human lung cancer cells (39), and promotes proliferation, reduces apoptosis, and facilitates glycolysis in colorectal cancer cells (40). Additionally, reports have demonstrated that miR147 directly targets Ndufa4 expression in osteoblasts (41), in macrophages (42), and in kidney cells (43). We show that miR147 regulates expression of Ndufa4 in IECs, while deletion of the miR147 binding site in the Ndufa4 3′-UTR abolishes this regulation, suggesting that Ndufa4 is a functionally regulated target of miR147 in IECs. Our study shows that expression of miR147 is absent in the stem cell/transit-amplifying cell compartment of the intestinal crypts in the colon but is profoundly increased in the differentiated cells, and this expression pattern correlates with the reported decrease in glycolytic metabolism in the differentiated epithelium (44) and with a decrease in Ndufa4 expression. Furthermore, it suggests that miR147-mediated reduction in Ndufa4 expression may offer a therapeutic opportunity in colorectal (and other) cancer treatment by overexpression of miR147 (utilizing miR147 mimetics), which would lead to a diminished capacity to utilize glycolysis by the tumor cells and to tumor cell death.

Hypoxia is a hallmark of inflamed intestinal tissues that influences immune responses and mucosal healing (45). Hypoxic conditions in the gut enhance the function of FOXP3-expressing regulatory T cells (Tregs), which are critical for maintaining immune tolerance and promoting epithelial repair. FOXP3, a key transcription factor in Tregs, is upregulated in response to hypoxia, thereby improving their immune regulatory function and promoting mucosal healing (46). Furthermore, we have recently demonstrated that miR-29a is negatively regulated by hypoxia-inducible factor 2α (HIF-2α) during intestinal inflammation (9), linking hypoxic conditions to miR-29a’s dysregulation, which can exacerbate inflammation and impair mucosal healing. Similarly, Yuan and Eltzschig (47) showed in a recent study that miR147, which is also induced by hypoxia, plays a protective role in the lung epithelium, reducing inflammation and enhancing barrier function. This suggests that miR147 may have a similar protective role in the intestinal epithelium under hypoxic conditions, further highlighting the importance of hypoxia-regulated miRNAs in mucosal healing. Together, miR147’s protective role represents critical mechanisms in maintaining epithelial integrity during inflammation, and their dysregulation may contribute to the impaired healing seen in IBD.

Ndufa4, a functionally regulated target of miR147 in IECs, modulates antiviral and inflammatory responses. Reduced Ndufa4 expression enhances type I interferon signaling, which restricts viral replication and supports mucosal protection during colitis (48–50). In miR147-deficient epithelia, elevated Ndufa4 levels correlate with reduced interferon response gene expression, suggesting that excessive Ndufa4 suppresses interferon signaling. miR147 is highly expressed in differentiated colonocytes, key producers of type I interferons during gut inflammation (51, 52) where it maintains low Ndufa4 levels to support robust interferon production. Additionally, colonocytes rely on oxidative phosphorylation for energy (3, 53). Overexpression of Ndufa4, a mitochondrial complex IV–associated protein, disrupts mitochondrial metabolism, impairing epithelial function during inflammation. Thus, miR147 promotes mucosal healing by repressing Ndufa4, ensuring metabolic homeostasis and effective interferon-mediated immunity in colitis.

In summary, our findings define a critical role for miR147 in maintaining intestinal epithelial integrity and facilitating mucosal repair through repression of Ndufa4. By modulating Ndufa4 expression to influence the metabolic profile and interferon responsiveness of differentiated colonocytes, miR147 emerges as a potential therapeutic target for both IBD and colorectal cancer.

Targeted delivery of miR147 mimics or Ndufa4-directed antisense oligonucleotides, either via aptamers, nanoparticles, or oral formulations, could potentially modulate epithelial metabolism and inflammation. These strategies, similar to miRNA-based therapies like miR-34a mimics (54), offer a precision medicine approach for treating inflammation-driven intestinal disease. Our findings establish miR147 as a pivotal regulator of intestinal homeostasis, offering what we believe is a novel therapeutic target for IBD and colorectal cancer. Future studies should focus on optimizing miR147-mimetic delivery systems and evaluating their efficacy in preclinical models.

## Methods

### Sex as a biological variable.

This study utilized male WT mice for all in vivo experiments, including DSS colitis models and spatial transcriptomics analyses (Figures 4, 6, and 7). Male mice were selected for their consistent susceptibility to DSS-induced colitis. All other mouse studies used both sexes, with sex largely not considered as a biological variable. The findings, including miR-147’s role in suppressing Ndufa4 to promote mucosal integrity and healing, are expected to apply to both sexes, as the miR-147/Ndufa4 axis is conserved in male and female IECs, supported by human single-cell data showing similar C15ORF48 expression patterns in both sexes (Supplemental Figure 10). Sex was not considered a biological variable for human single-cell data analyses, as these were aggregated from public repositories without sex-specific stratification.

### Mice.

WT, *CMV Cre* [B6.C-Tg(CMV-cre)1Cgn/J], and *Villin Cre* mice [B6.Cg-Tg(Vil1-cre)997Gum/J] were obtained from The Jackson Laboratory. *miR147^fl/fl^* *Villin Cre^+^* mice were generated by breeding *miR147^fl/fl^* mice (55) with *Villin Cre* mice to achieve IEC-specific deletion of *miR147*. *miR147CMV* (*miR147^–/–^*) mice were generated by crossing *miR147^fl/fl^* mice with *CMV Cre* mice (The Jackson Laboratory) to delete the *miR147* sequence within the *AA467197* gene via Cre-lox recombination, followed by removal of the *Cre* allele through backcrossing. *Ndufa/mir147del* mice were a gift from Nathenial Berg and Xiaoyi Yuan (The University of Texas Health Science Center at Houston) and bred at our facility. Briefly, the 3′-UTR binding site of miR147 in the *Ndufa4* locus was flanked with *loxP* sites to allow for tissue-specific deletion. Mice were crossed with *CMV Cre* and whole-body deletion was produced and used in all experiments. Germ-free WT mice were sourced from the Baylor College of Medicine Gnotobiotic Core. *miR147tdTomato* reporter mice were engineered by inserting a *tdTomato* gene preceded by an internal ribosomal entry site (IRES) immediately following the stop codon of the *AA467197* gene and preceding the *miR147* sequence, enabling polycistronic expression from the *AA467197* promoter (schematic in Figure 1B). tdTomato fluorescence was imaged and quantified using the IVIS imaging system (Caliper/PerkinElmer).

Mice were housed under specific pathogen–free conditions at 20°C–26°C with 30%–70% humidity and a 12-hour light/dark cycle. Male and female mice aged 8–12 weeks were used for all experiments, except for DSS colitis studies, which used only male mice due to their greater disease sensitivity, reducing the required cohort size.

### Tissue and cell isolation.

Colon and cecum epithelial cells: Colons and ceca were excised, flushed with ice-cold Hanks’ balanced salt solution (HBSS; H6648-1L, Sigma-Aldrich), and cut open longitudinally. Tissues were incubated in 2 mL Cell Stripper buffer (25-056-CI, Corning) at 37°C for 30 minutes with gentle shaking every 5 minutes to release crypts and IECs. Liberated IECs and crypts were pelleted at 500*g* for 5 minutes at 4°C, washed twice with ice-cold HBSS, and used for RNA extraction, flow cytometry, Western blot, or organoid culture. For overnight culture, IECs were plated in DMEM supplemented with 50 ng/mL epidermal growth factor (EGF) and 5% fetal bovine serum (FBS) at 37°C with 5% CO_2_.

Colon LPLs: LPLs were isolated from colons of 8- to 12-week-old WT mice using the Lamina Propria Dissociation Kit (130-097-410, Miltenyi Biotec) per the manufacturer’s instructions. Cell pellets were lysed in QIAzol (79306, QIAGEN) for RNA extraction.

### Organoid culture.

Colonic and cecal crypts were isolated as described above, incubated in Cell Stripper buffer at 4°C for 30 minutes on a rocking platform, and washed in 4 changes of HBSS at 300*g*, 250*g*, 220*g*, and 200*g* to remove single cells. Crypts were plated in Matrigel domes (356237, Corning) and cultured in Growth Media containing EGF, Noggin, R-spondin1, and Wnt3a (Baylor College of Medicine Organoid Core). Media were refreshed every 3 days. Organoid growth and survival were monitored with an Olympus CKX53 inverted microscope equipped with a light source U-HGLG (Olympus).

### RNA extraction and qPCR.

IECs or tissues were lysed in QIAzol for RNA extraction. RNA was isolated using chloroform (60051, Calbiochem) for phase separation, precipitated with isopropanol (222218, Thermo Fisher Scientific), washed twice in 70% ethanol, and dried using an Eppendorf Vacufuge Plus (D-AL settings) for 3 minutes. RNA pellets were dissolved in DEPC-treated water and quantified. Total RNA was reverse transcribed to cDNA using the High-Capacity cDNA Reverse Transcription Kit (Applied Biosystems/Thermo Fisher Scientific). qPCR was performed using TaqMan Gene Expression Assays (FAM) for 18s (4351368, assay ID: Hs99999901_s1), Ndufa4 (4331182, assay ID: Mm00809672_s1), and AA467197/Nmes1 (4351372, assay ID: Mm01268692_m1), and TaqMan MicroRNA Assays for miR147 (4440887, assay ID: 002262) and U6 snoRNA (4440888, assay ID: 001973) (all from Thermo Fisher Scientific). Relative gene expression was normalized to 18s (for mRNA) or U6 snoRNA (for miRNA) using the ΔΔCt method.

### RNA-seq.

Colonic IECs from *Villin Cre^+^* and *miR147^fl/fl^ Villin Cre^+^* mice treated with 3% DSS (36–50 kDa, Sigma-Aldrich) in drinking water for 5 days, followed by 1 day of regular water, were isolated as described in *Tissue and cell isolation*. RNA was extracted using the TRIzol method, quantified, and quality-checked with a NanoDrop spectrophotometer. Library preparation was performed using the NEBNext Ultra II RNA Library Prep Kit (E7770, New England Biolabs), and sequencing was conducted on an Illumina NovaSeq 6000 (150-bp paired-end reads, ~30 million reads/sample) at the UTHealth Genomic Core. Data were preprocessed with FastQC (https://www.bioinformatics.babraham.ac.uk/projects/fastqc/) for quality control, aligned to the mouse genome (mm10) with STAR (http://code.google.com/p/rna-star/), and differential expression analyzed using DESeq2 (https://bioconductor.org/packages/release/bioc/html/DESeq2.html). Results were visualized in GraphPad Prism (version 10.1.1).

### Flow cytometry.

IECs from colons and ceca were isolated as described in *Tissue and cell isolation*, incubated with anti-CD16/CD32 (1:10; clone 93, 101302, BioLegend) for Fc blocking, and stained with a LIVE/DEAD Fixable Near-IR Dead Cell Stain Kit (L10119, Invitrogen), anti-CD326 (EpCAM; 1:200; clone G8.8, 118225, BioLegend), and anti-CD44 (1:200; clone IM7, 103011, BioLegend). Events were acquired on a CytoFLEX flow cytometer (Beckman Coulter) and analyzed using FlowJo (version 10). Gating strategies are shown in Supplemental Figure 3B.

### Spatial transcriptomics.

Colonic tissues from healthy (n981_healthy; 386,865 pseudo-cells) and DSS-treated (n2_DSS; 421,574 pseudo-cells; 3% DSS for 3 days) mice were cleaned of adipose tissue, cut longitudinally, and flushed with cold PBS. Tissues were rolled into Swiss rolls (distal colon at the center, proximal colon outer), fixed in 10% formalin for 16 hours, and embedded in paraffin. Sections (4 μm) were cut using a microtome, placed on SuperFrost Plus slides (12-550-15, Thermo Fisher Scientific), and processed per the Visium HD FFPE Tissue Preparation Handbook (10x Genomics). After deparaffinization, H&E staining, and imaging, probe hybridization, ligation, slide preparation, probe release, extension, and library construction were performed following the Visium HD Spatial Gene Expression Reagent Kits User Guide. Sequencing was conducted on an Illumina NovaSeq 6000 (43 bp read 1, 50 bp read 2, 10 bp i7/i5 sample indexes). Data were quality filtered (gene count: 10–1,600; UMI count: 10–2,200; mitochondrial fraction: <10%) and analyzed using non-negative matrix factorization (NMF), principal component analysis (PCA), and UMAP in Seurat (https://github.com/satijalab/seurat). Non-negative least squares (NNLS) deconvolution was performed using Semla’s RunNNLS() to map human cell-type signatures (24) onto murine data, with spatial feature maps generated using MapFeatures/MapMultipleFeatures.

### Human single-cell data analysis.

The Pan-GI Cell Atlas (25) (dataset: 1_Healthy_Pan-GI_atlas_all_lineages_2025.h5ad) was used as a reference for healthy human gastrointestinal single-cell RNA-seq data. Data were processed using Scanpy (v1.9+) in Python (https://scanpy.readthedocs.io/en/1.9.x/). Expression of C15ORF48 and NDUFA4 was visualized via UMAP embeddings, colored by normalized expression, using louvain_annot or level_3_annot annotations. Average expression and percentage of cells expressing target genes were computed across epithelial cell types (e.g., enterocytes, goblet cells, BEST4^+^ cells) and visualized using dot plots (hires_DotPlot_Epithelial_C15ORF48_NDUFA4.png/svg). Gene expression was standardized, and results were exported as high-resolution.png and.svg files.

### Fecal microbiome sequencing.

Transnetyx services were utilized for microbiome sequencing in mice. This approach utilizes shallow shotgun, next-generation sequencing and the One Codex analysis platform for taxonomic resolution. Mouse feces were collected into Transnetyx storage vials and shipped according to the service provider recommendations.

### DSS colitis.

Mice were administered 3% or 3.5% DSS (36–50 kDa, Sigma-Aldrich) in drinking water for 5 days, followed by regular water for 1–2 days, as indicated; 3.5% DSS was used for in vivo phenotype assessments, while 3% DSS was used for IEC collection experiments to minimize rapid epithelial loss and ensure cell viability for molecular analyses. Body weight was monitored daily. On day 7 (or day 4 for some experiments), mice were euthanized, and colons were measured for length and weight. Colonic IECs were isolated for RNA extraction, qPCR, or Western blot analysis. Tissues were processed for histopathology.

### FITC-dextran permeability assay.

Mice were fasted for 4 hours and gavaged with 100 μL FITC-dextran (600 mg/kg; 4,000 kDa, Sigma-Aldrich) in randomized order. Four hours later, mice were anesthetized with isoflurane (2%–3% in oxygen) and retro-orbital blood collected. Serum was separated by centrifugation at 10,000*g* for 10 minutes and analyzed in duplicate for FITC-dextran fluorescence using a BioTek microplate reader (excitation: 485 nm, emission: 535 nm).

### Histology.

Colons were excised, opened longitudinally, flushed with ice-cold PBS (P3813, Sigma-Aldrich), and fixed in 10% neutral buffered formalin (HT501128, Sigma-Aldrich) for 24 hours at room temperature. Tissues were dehydrated through a graded ethanol series, cleared in xylene, and embedded in paraffin. Sections (5 μm) were cut using a microtome, mounted on SuperFrost Plus slides, and stained with hematoxylin and eosin (H&E) using standard protocols. Slides were imaged using a Leica microscope with LAS X Life Science software.

Histological assessment of colitis pathology was performed in a blinded manner by 2 independent investigators, following criteria adapted from Tiwari-Heckler et al. (50). Three parameters — immune cell infiltration, epithelial damage, and mucosal architecture disruption — were scored separately for the proximal, medial, and distal colon. Scores for each parameter were averaged across the 3 regions to yield a composite inflammation score per animal (range: 0–12).

### Cryosectioning and immunofluorescence.

Colons were flushed with HBSS, fixed in 4% paraformaldehyde (W14G514, Alfa Aesar) for 30 minutes at room temperature, and incubated in 30% sucrose in PBS overnight until tissues sank. Swiss rolls were embedded in Frozen Section Compound (FSC 22 Clear, 3801480, Leica), solidified on dry ice, and stored at –80°C. Cryosections (8 μm) were cut, fixed in cold acetone (>99.5%) for 10 minutes, air-dried for 20 minutes, and blocked with 10% donkey serum in 1% PBS/0.5% Tween 20 for 1 hour. Sections were incubated with primary antibodies (goat anti-tdTomato, 1:200, LS-C340696, LSBio; anti-NDUFA4 rabbit, 1:1000, ab129752, Abcam) for 1 hour, followed by secondary antibodies (donkey anti-goat Alexa Fluor 488, 1:400, A32814; donkey anti-rabbit Alexa Fluor 647, 1:400, A32795; Thermo Fisher Scientific) for 1 hour or WGA (Invitrogen, W32466; Alexa Fluor 647) for 3 minutes. Slides were mounted with Vectashield Antifade Mounting Medium with DAPI (H-1200, Vector Laboratories) and incubated overnight at 4°C before imaging on a Leica confocal microscope.

### Western blot.

IECs were isolated using Cell Stripper, pelleted at 500*g* at 4°C, washed twice in PBS, and flash-frozen in liquid nitrogen. Proteins were extracted in RIPA buffer with protease and phosphatase inhibitors, incubated on ice for 20 minutes, and centrifuged at 13,000*g* for 20 minutes at 4°C. Supernatants were quantified using the Coomassie Protein Assay (1856209, Thermo Fisher Scientific). Proteins were separated on 4%–20% Mini-PROTEAN TGX gels (4568096, Bio-Rad), transferred to 0.2 μm PVDF membranes (1704156EDU, Bio-Rad), and blocked with 5% skim milk or EveryBlot Blocking Buffer (12010020, Bio-Rad). Membranes were incubated overnight at 4°C with primary antibodies (rabbit anti-NMES1, 1:500, ab128382, Abcam; rabbit anti-NDUFA4, 1:1000, ab129752, Abcam; rabbit anti–α-tubulin, 1:1000, 2144S, Cell Signaling Technology), followed by anti-rabbit IgG HRP-linked secondary antibody (1:3000, 7074S, Cell Signaling Technology) for 1 hour at room temperature. Images were captured using a ChemiDoc Touch Imaging System (version 2.3.0.07, Bio-Rad) and quantified with ImageJ (NIH), normalized to α-tubulin.

### Statistics.

Mice were randomly assigned to experimental groups. Male and female mice were used for all studies except DSS colitis, where only males were used due to greater disease sensitivity. Histological scoring was performed by researchers blinded to sample identity. Statistical analyses were conducted using GraphPad Prism (version 10.1), with specific tests (*t* test, 1-way ANOVA) indicated in figure legends. Data are expressed as mean ± SEM. Statistical significance was set at *P* less than 0.05. Two-tailed *t* tests were used in all figure panels, except for Figure 6, M and N, where 1-tailed tests were used (Welch’s *t* test for M, unpaired *t* test for N) based on the directional hypothesis that Ndufa4 expression and protein abundance would increase in *Ndufa4/miR147del* mice due to the deletion of the miR147 binding site in the Ndufa4 3′-UTR. This hypothesis was supported by prior evidence showing upregulated Ndufa4 expression in miR147-deficient IECs (Figure 5B and Figure 6, C–H).

Sample sizes were determined through power analysis to ensure adequate power for detecting biologically meaningful differences. For DSS colitis experiments (e.g., weight loss, colon length, histological scores; Figures 4 and 6), calculations were based on preliminary data. Assuming a 2-tailed *t* test, an effect size of 1.2 (based on expected colon length differences between WT and miR147-deficient mice), a standard deviation of 10% of the mean, α = 0.05, and power (1 – β) = 0.80, a minimum of 8 mice per group was required. For ANOVA-based comparisons (e.g., Figure 4H), an effect size *f* = 0.4, α = 0.05, and power = 0.80 yielded a minimum of 10 mice per group. Sample sizes of 8–11 mice per group were used for DSS colitis experiments, meeting or exceeding requirements. For other experiments (e.g., qPCR, flow cytometry; Figures 1–3), sample sizes of 2–4 mice per group were based on prior studies showing low variability in molecular endpoints, with post hoc confirmation of power greater than 0.80. Floating bar graphs were used to visualize data ranges, with each bar spanning from the minimum to the maximum values of the dataset for each group. The bar represents the full range of data, with a line or marker indicating the mean. Individual data points were overlaid. Analyses were performed using GraphPad Prism (v10.3).

### Study approval.

All animal experiments were conducted in accordance with University of Texas Health Science Center (UTHealth) institutional guidelines and approved by the UTHealth Institutional Animal Care and Use Committee. Human single-cell data were obtained from the publicly available Pan-GI Cell Atlas, and no additional human study approval or informed consent was required for this secondary analysis.

### Data availability.

Values for all data points are reported in the Supporting Data Values file. Supporting data for Figure 5 and Supplemental Figure 4 are included in Supplemental Dataset 1 and Dataset 2, respectively. Raw sequencing data for the spatial transcriptomics study are available at the NCBI Sequence Read Archive (SRA) under BioProject accession PRJNA1284556 (https://www.ncbi.nlm.nih.gov/sra/PRJNA1284556).

## Author contributions

AKC and HKE conceptualized the study. AKC, CHT, ZZ, and HKE deveoped methodology. AKC, AD, CHT, VV, XH, NVP, NFM, JW, and HL conducted experiments. AKC, XH, and HKE wrote the manuscript. AKC, CHT, NM, XY, KF, and AD reviewed and edited the manuscript. HL, ZZ, XY, and HKE provided resources. AKC and HKE provided supervision and project administration.

## Funding support

Assistant Secretary of Defense for Health Affairs (endorsed by the Department of Defense, through the Peer Reviewed Medical Research Program), award no. HT9425-23-1-0094, to AKC.

Paula Mischer Foundation, grant, to AKC.

Jerold B. Katz Foundation, grant, to AKC and HKE.

NIH, grants R01-HL154720, R01-DK122796, and R01-HL133900, to HKE.

Crohn’s and Colitis Foundation of America, grant, to HKE.

NIH, grant R01-HL155950, to XY.

Parker B. Francis Fellowship, to XY.

American Lung Association Catalyst Award, CA-622265, to XY.

## Supplementary Material

Supplemental data

Supplemental data set 1

Supplemental data set 2

Unedited blot and gel images

Supplemental video 1

Supplemental video 2

Supplemental video 3

Supplemental video 4

Supplemental video 5

Supporting data values

## Version 1 09/16/2025

In-Press Preview

## Version 2 10/22/2025

Electronic publication
