# Supplementary material for: miR147 promotes mucosal integrity and healing in intestinal inflammation
Source: JCI Insight. 2025 Sep 16;10(20):e190466. doi: 10.1172/jci.insight.190466 (PMC12581662; doi:10.1172/jci.insight.190466)
Supplement: Supplemental data [file jciinsight-10-190466-s071.pdf]

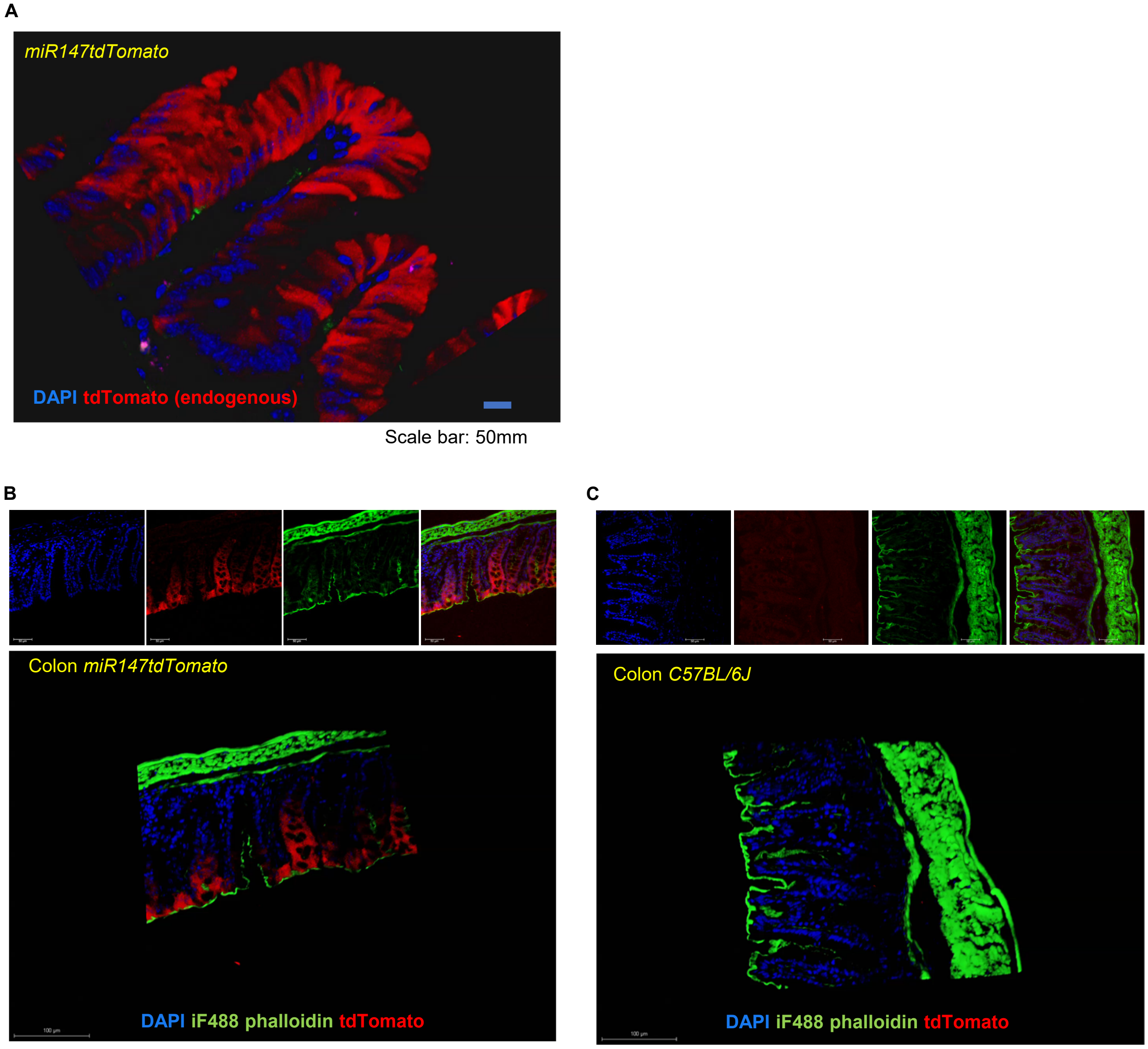

Supplemental figure 1. 3D confocal imaging of miR147tdTomato reporter expression in intestinal epithelium

Panel (**A**) shows a still image from Supplemental Video 1 of cecal tissue from *miR147tdTomato* reporter mice stained with DAPI (H-1200, Vector Laboratories), highlighting miR147tdTomato expression in the luminal portion of crypts. See also Supplemental Video 1 for the full 3D projection. Panel (**B**) shows a still image from Supplemental Video 2 of colonic tissue from *miR147tdTomato* reporter mice stained with Alexa Fluor 488-conjugated phalloidin (A12379, Invitrogen; F-actin) and DAPI, highlighting luminal epithelial expression. See also Supplemental Video 2. Panel (**C**) shows a still image from Supplemental Video 3 of colonic tissue from control *C57BL/6J* mice stained as in B, showing no tdTomato signal. See also Supplemental Video 3. Scale bar: 50 μm. Experiments were repeated three times.

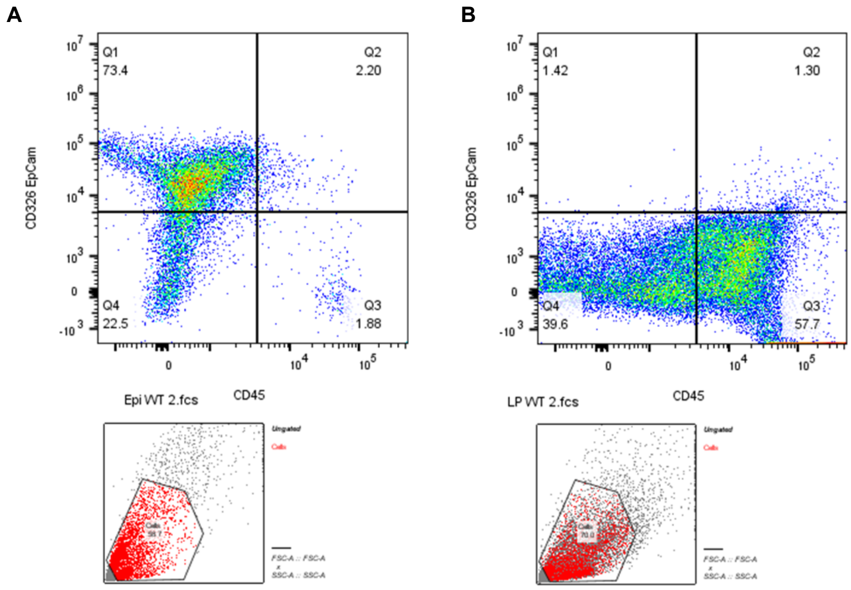

Supplemental Figure 2. Fractionation and flow cytometry analysis of mouse colon
Colons from *C57BL/6J* mice were excised, opened longitudinally, flushed with phosphate-buffered saline (PBS; P3813, Sigma-Aldrich), and processed for cell fractionation. (**A**) Epithelial cell fraction isolated using Cell Stripper (25-056-CI, Corning) and analyzed by flow cytometry. (**B**) Remaining tissue digested using the Lamina Propria Dissociation Kit (130-097-410, Miltenyi Biotec), followed by flow cytometry analysis. Back-gating strategies are shown below each panel.

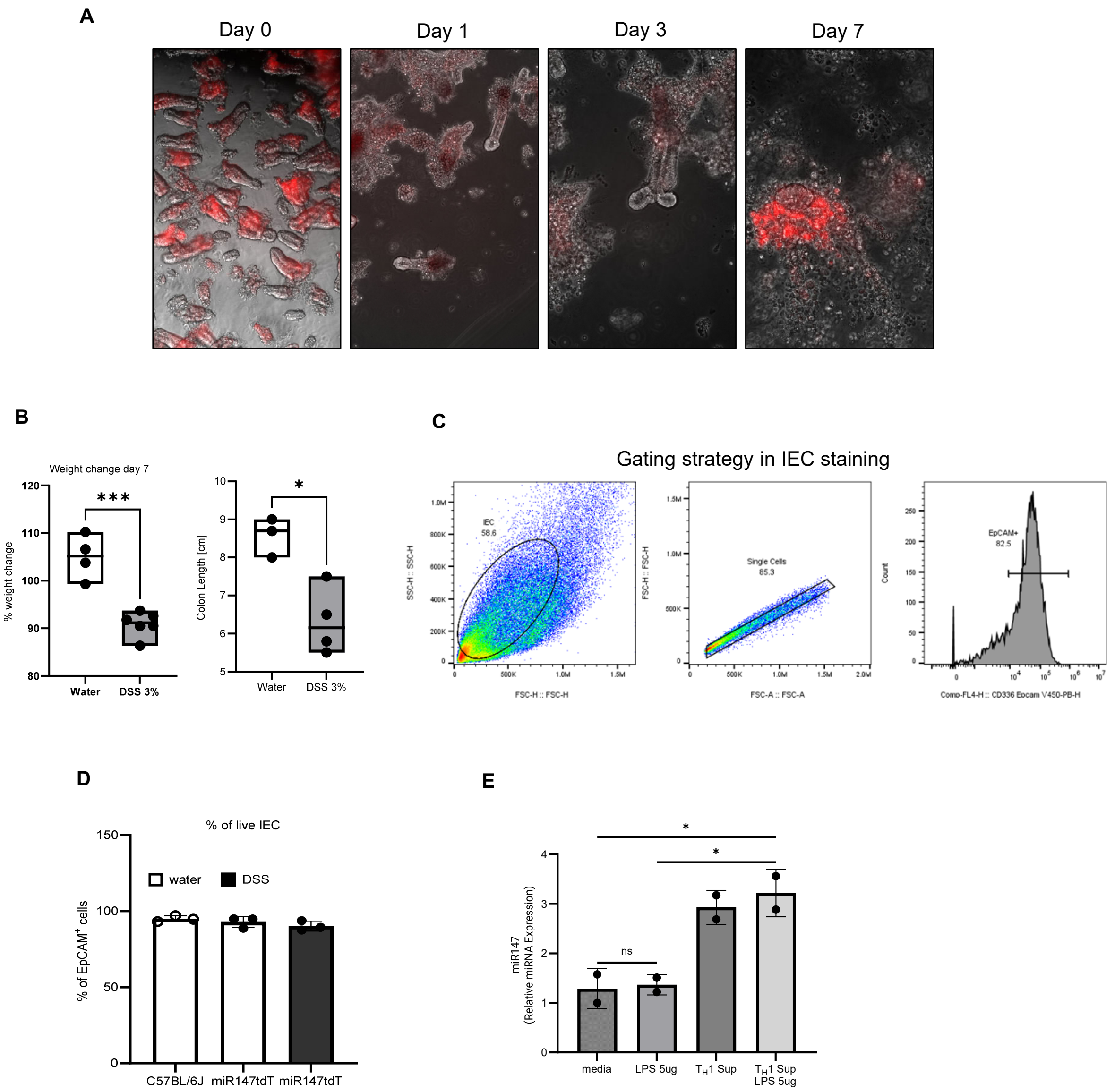

Supplemental Figure 3. miR147tdTomato reporter expression in organoids and during inflammation

Panel (**A**) shows a still image from Supplemental Video 4 of intestinal organoids derived from *miR147tdTomato* reporter mice cultured in Matrigel domes (356237, Corning). Images include freshly isolated crypts (Day 0), organoid growth (Days 1–7), and tdTomato expression in differentiated cells in the central portion of organoids (Day 7). See also Supplemental Video 4 for full organoid growth. (**B**) DSS colitis weight and colon length changes for *miR147tdTomato* mice. (**C**) Gating strategy for identifying intestinal epithelial cells (IECs) in flow cytometry experiments. (**D**) Proportion of live IECs in colonic samples from *miR147tdTomato* mice treated with 3% dextran sulfate sodium (DSS; 36–50 kDa, Sigma-Aldrich) for 5 days or untreated controls, quantified by flow cytometry (n=3 mice/group). (**E**) miR147 expression in IECs treated with indicated stimuli, measured by qPCR (n=2 mice/group). Data in (**B, D, E**) are expressed as mean ± S.E.M.; (**B**) unpaired two-tailed t-test, (**D, E**) one-way ANOVA, $p < 0.05$. Male and female mice were used.

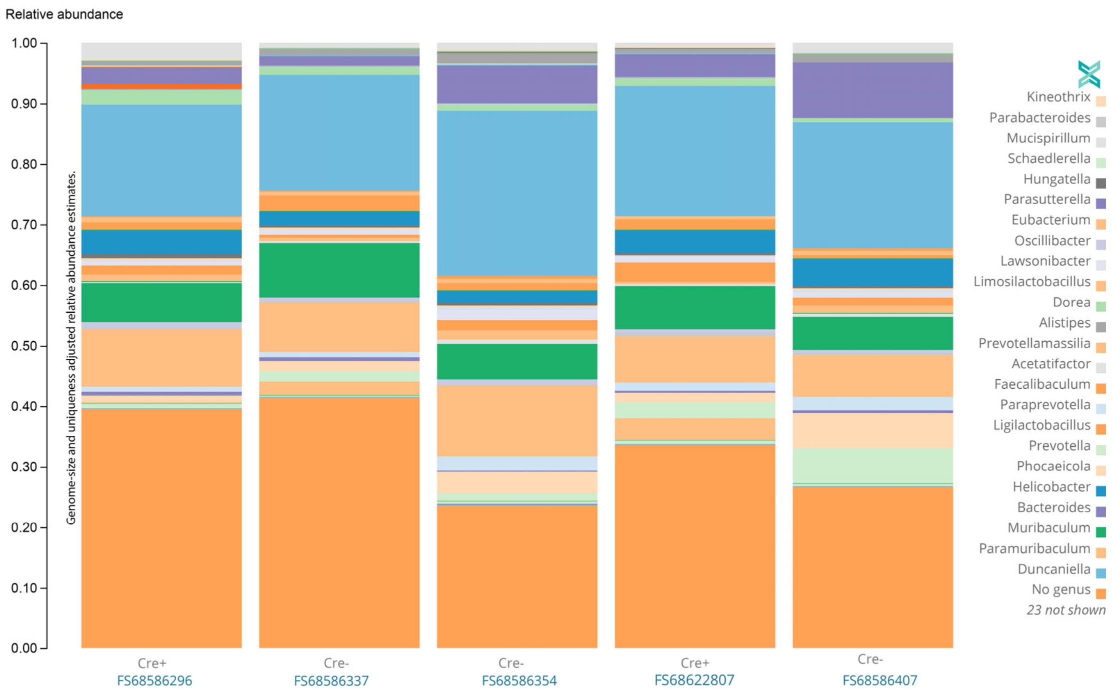

Supplemental Figure 4. Microbiome composition in miR147-deficient intestinal epithelium
Fecal samples from *miR147loxP/loxPVillinCre*+ and co-housed *miR147loxP/loxPVillinCre*- littermates were sequenced by Transnetyx Microbiome Services. Genus-level microbial abundance is shown for five male littermates, with no significant differences observed. Experiment was performed once.

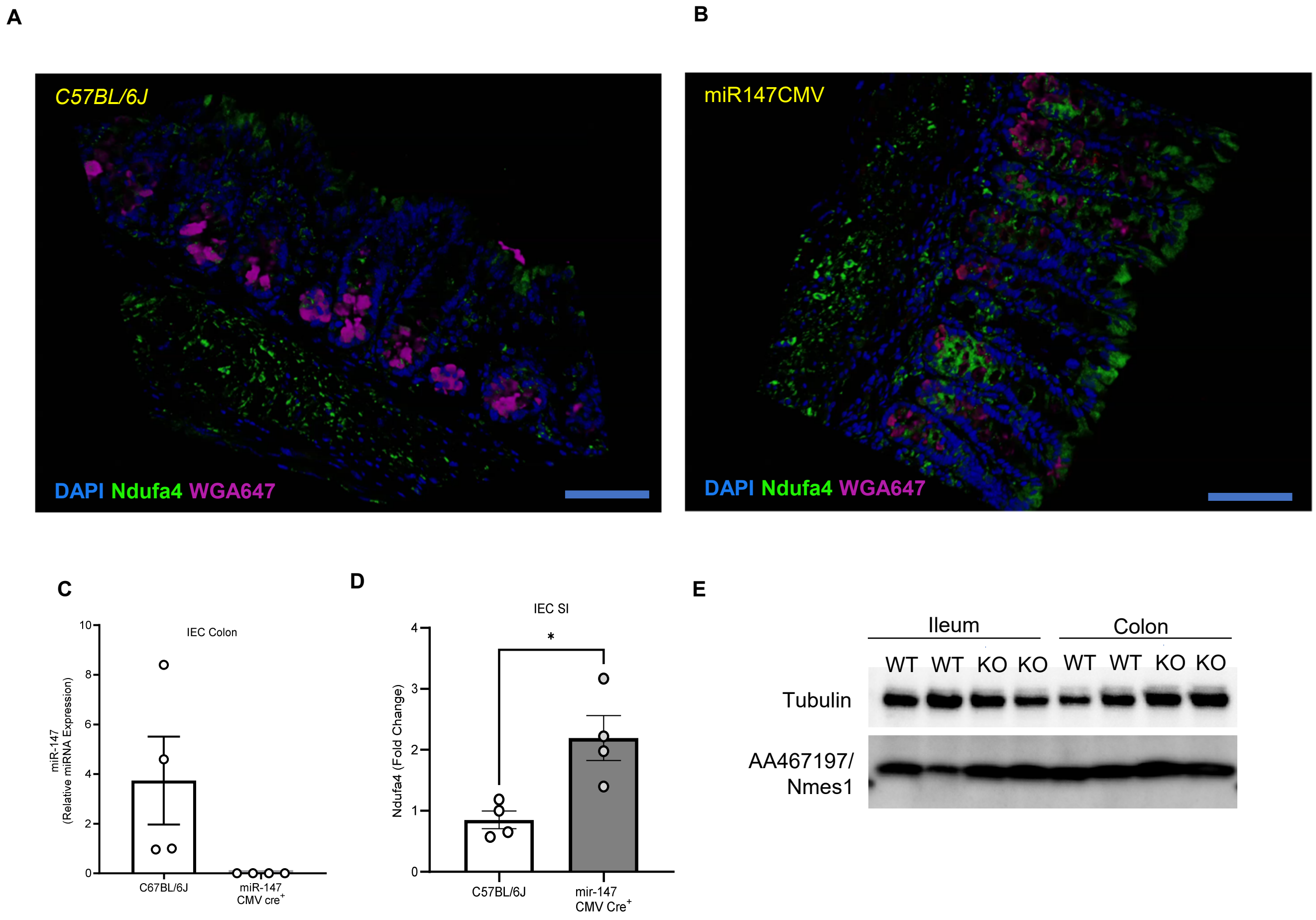

Supplemental Figure 5. Ndufa4 and AA467197 (Nmes1) expression in miR147-deficient intestinal epithelium

Panel (**A**) shows a still image from Supplemental Video 5 of frozen cecal sections from WT mice stained with anti-Ndufa4 antibody (rabbit polyclonal, ab129752, Abcam), donkey anti-rabbit Alexa Fluor 488 secondary antibody (A32790, Invitrogen), DAPI (H-1200, Vector Laboratories; nuclei), and Wheat Germ Agglutinin (WGA, Alexa Fluor 647, W32466, Invitrogen; mucus granules). See also Supplemental Video 5. Panel (**B**) shows a still image from Supplemental Video 6 of frozen cecal sections from miR147CMV mice stained as in A. See also Supplemental Video 6. (**C-D**) RNA extracted from isolated colonic and small intestinal IECs from miR147CMV and WT mice (n=4 mice/group), with expression of (**C**) miR147 and (**D**) Ndufa4 measured by qPCR. (**E**) Protein extracts from ileal and colonic IECs from miR147CMV and WT mice analyzed by western blot using anti-NMES1 antibody (ab128382, Abcam). Data in (**C-D**) are expressed as mean ± S.E.M.; unpaired two-tailed t-test, $p < 0.05$.

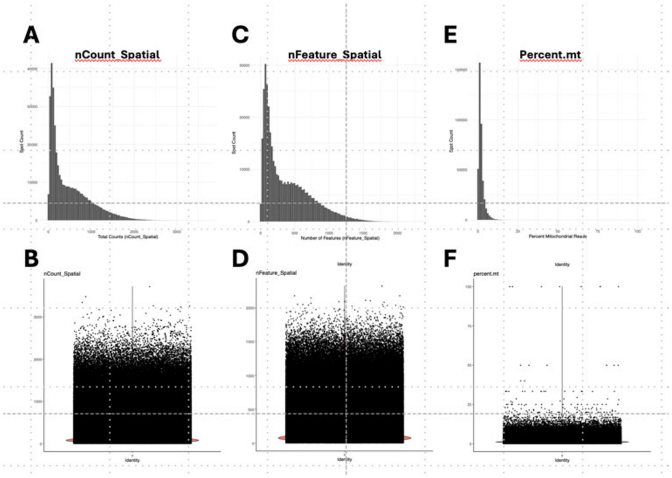

Supplemental Figure 6. Quality control metrics for spatial transcriptomics of n981_healthy
Pre-quality control (QC) metrics for the n981_healthy murine colon sample (386,865 pseudo-cells, 8-μm bins). (**A-B**) Total number of molecules detected per pseudo-cell. (**C-D**) Number of unique genes detected per pseudo-cell. (**E-F**) Mitochondrial content per pseudo-cell. Data were generated using Visium HD spatial transcriptomics.

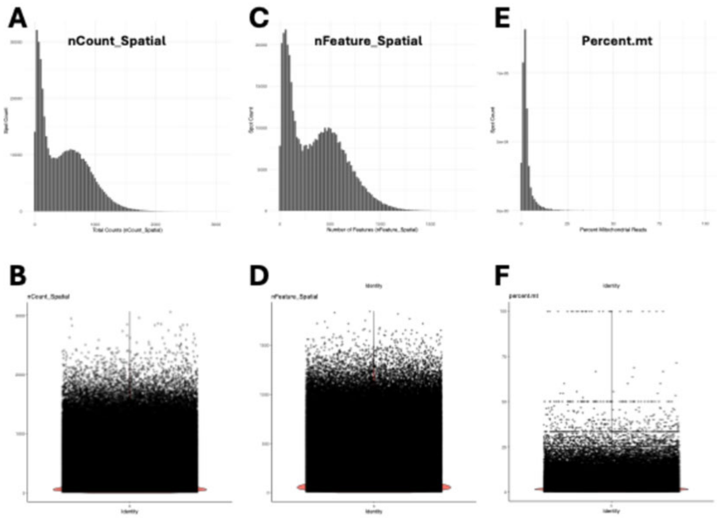

Supplemental Figure 7. Quality control metrics for spatial transcriptomics of n2_DSS
Pre-quality control (QC) metrics for the n2_DSS murine colon sample (421,574 pseudo-cells, 8-μm bins). (**A-B**) Total number of molecules detected per pseudo-cell. (**C-D**) Number of unique genes detected per pseudo-cell. (**E-F**) Mitochondrial content per pseudo-cell. Data were generated using Visium HD spatial transcriptomics.

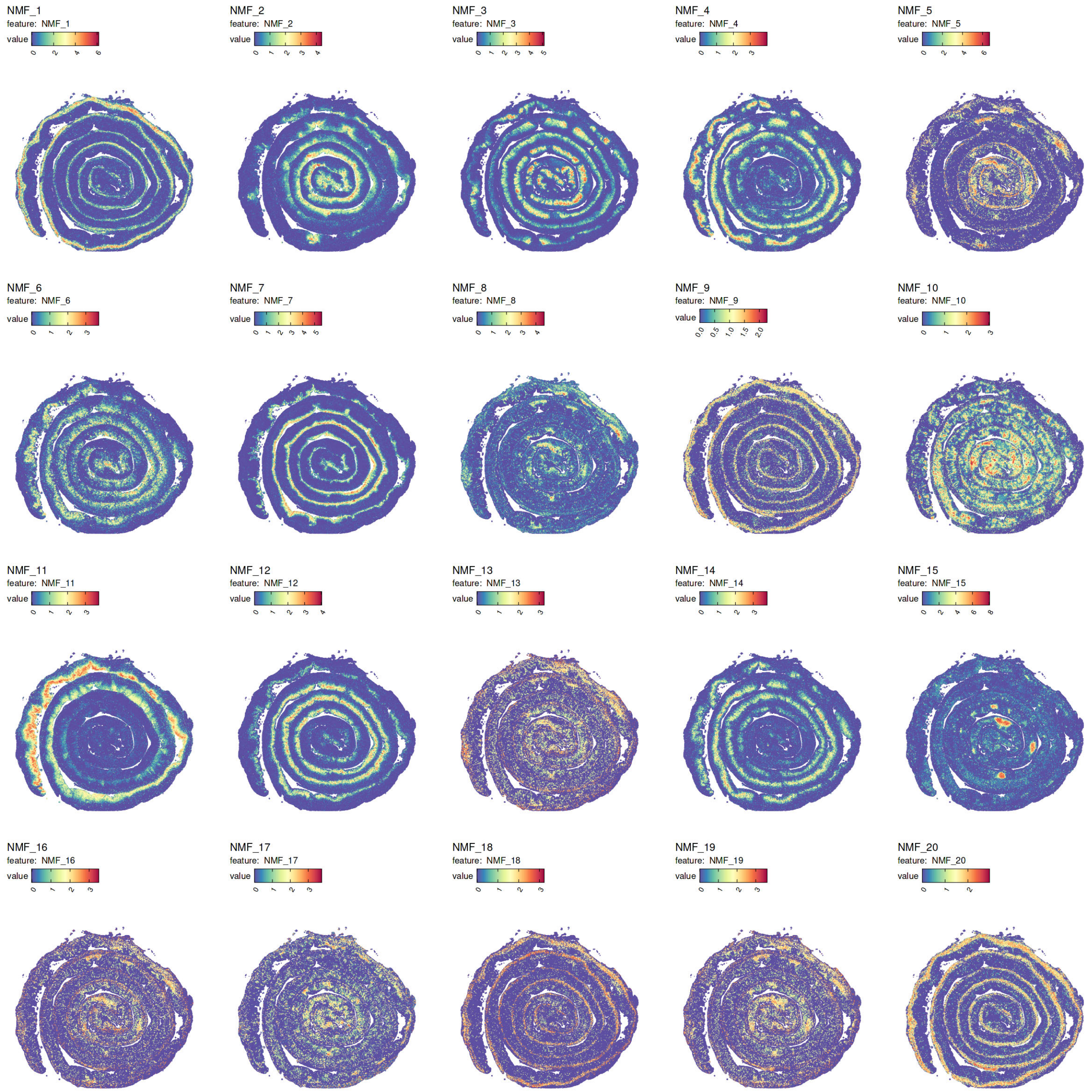

Supplemental Figure 8. Spatial distribution of transcriptomic factors in healthy murine colon
Spatial distribution of 20 transcriptomic factors in the n981_healthy murine colon sample, analyzed using Visium HD spatial transcriptomics. Factors were identified through non-negative matrix factorization (NMF) and mapped to 8-μm bins. Data were visualized using Seurat against H&E-stained sections.

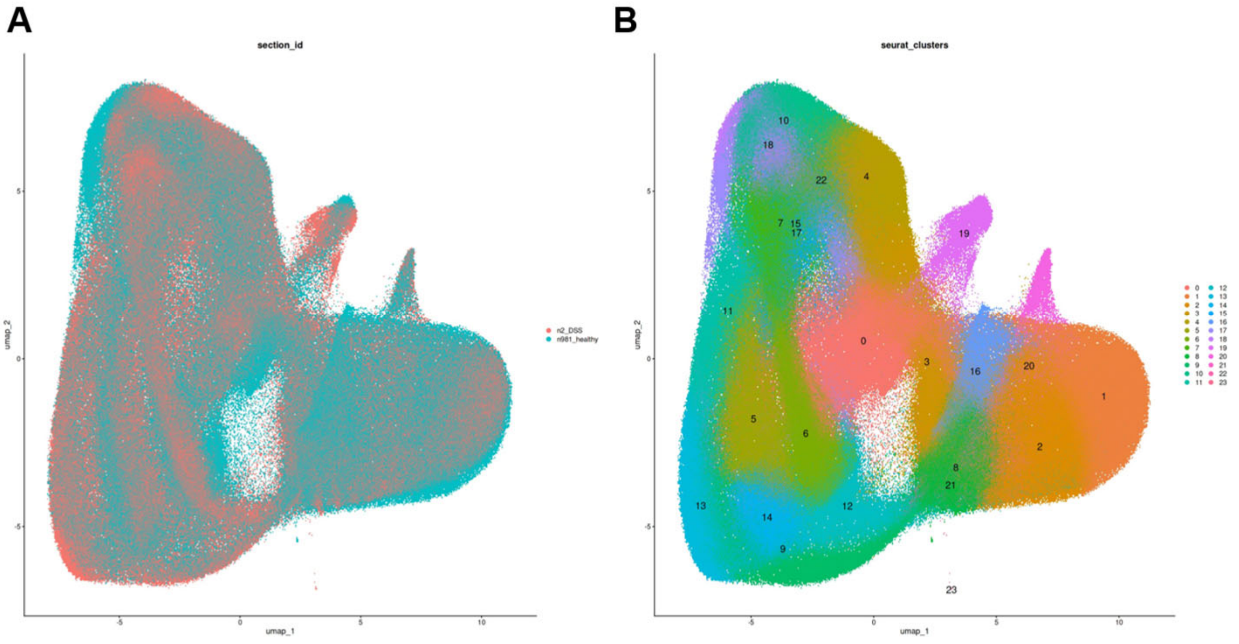

Supplemental Figure 9. UMAP embedding of integrated colonic single-cell transcriptomes
(**A**) UMAP plot of integrated single-cell transcriptomes from n981_healthy and n2_DSS (3% DSS-treated, 36–50 kDa) murine colon samples, colored by sample origin, demonstrating successful data integration. (**B**) UMAP plot colored by 23 transcriptomic clusters, representing distinct cell types or states identified post-integration. Analysis was performed using Seurat.

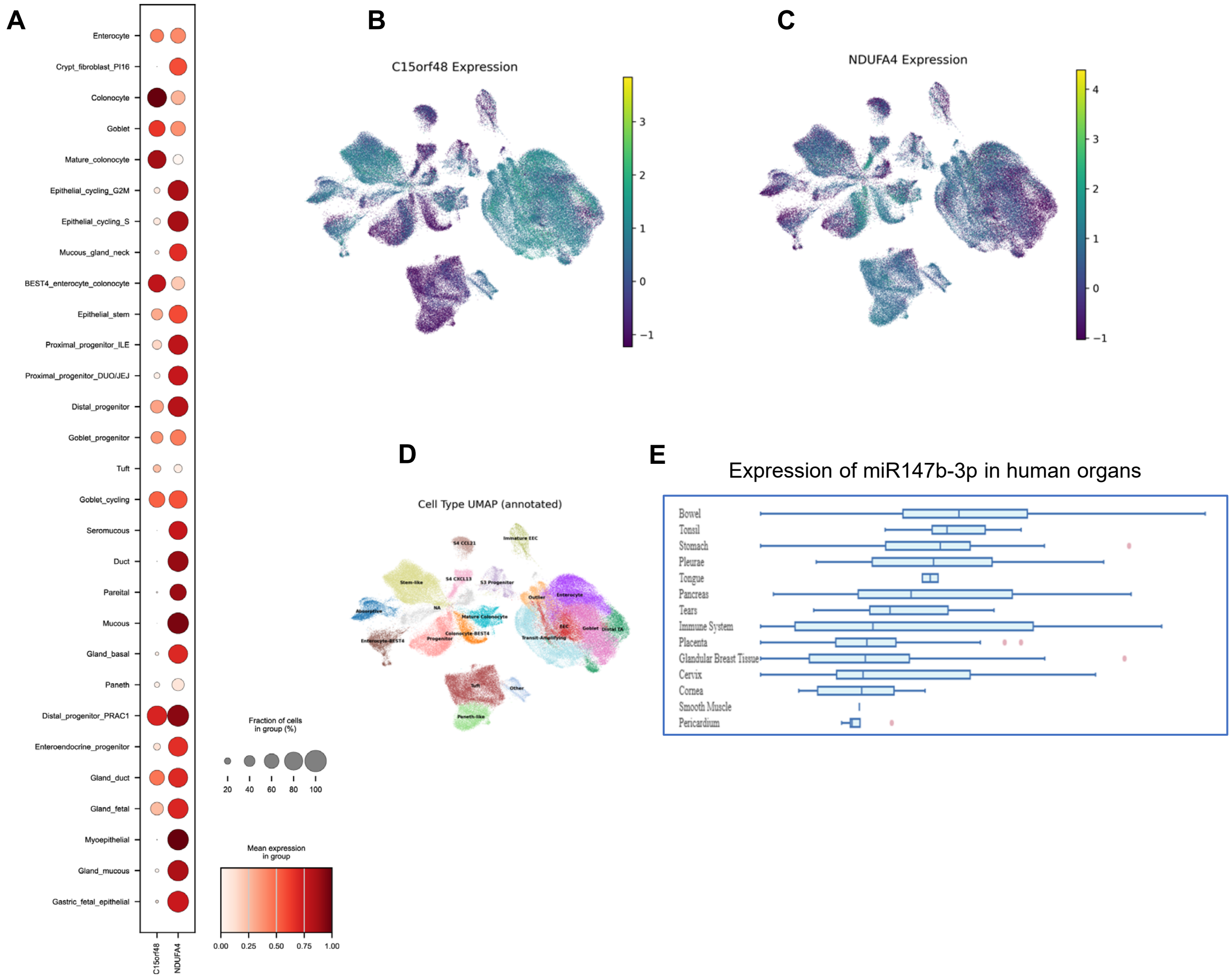

Supplemental Figure 10. Single-cell transcriptomic analysis of C15ORF48 (NMES1) and NDUFA4 in human gastrointestinal epithelium

(**A**) Dot plot of C15ORF48 (NMES1) and NDUFA4 expression across epithelial subtypes in the healthy human gastrointestinal tract, derived from the Pan-GI Cell Atlas (1_Healthy_Pan-GI_atlas_all_lineages_2025.h5ad). Epithelial cells were identified by keyword matching in level_3 annotations. Scaled average expression (color intensity) and fraction of expressing cells (dot size) were computed using Scanpy (v1.9+, standard_scale="var"). (**B**) UMAP visualization of human intestinal epithelial single-cell transcriptomes, colored by C15ORF48 expression (Viridis colormap), clustered using the Louvain algorithm with level_3 annotations. (**C**) UMAP visualization of NDUFA4 expression in epithelial cells, processed as in B. (**D**) UMAP embedding of epithelial cells, annotated by Louvain clustering and curated level_3 cell type definitions (e.g., enterocyte, goblet, stem-like), with colors representing distinct subtypes. (**E**) Ranked expression levels of miR147 in human organs, retrieved from the miRNA Human Tissue Atlas (https://ccb-compute2.cs.uni-saarland.de/mirnatissueatlas_2025). Data in (**A-D**) were normalized and analyzed using Scanpy.
