# Supplementary figures and images for: miR147 promotes mucosal integrity and healing in intestinal inflammation
Source: JCI Insight. 2025 Sep 16;10(20):e190466. doi: 10.1172/jci.insight.190466 (PMC12581662; doi:10.1172/jci.insight.190466)

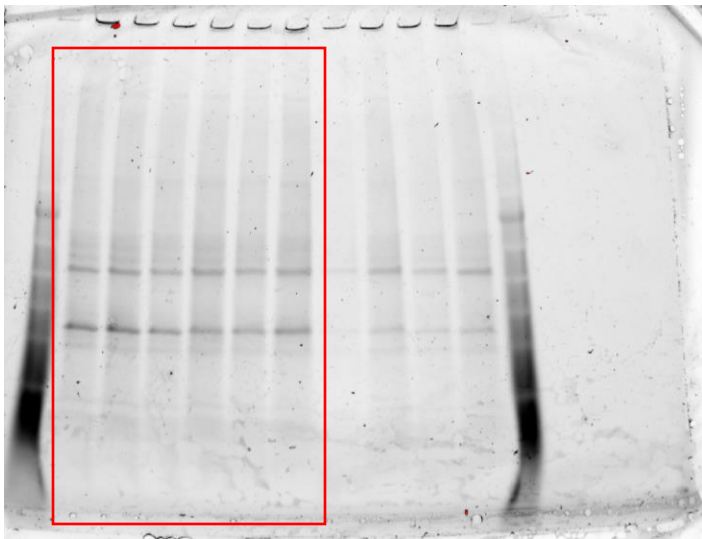

Unaltered Western blot images for Figure 60

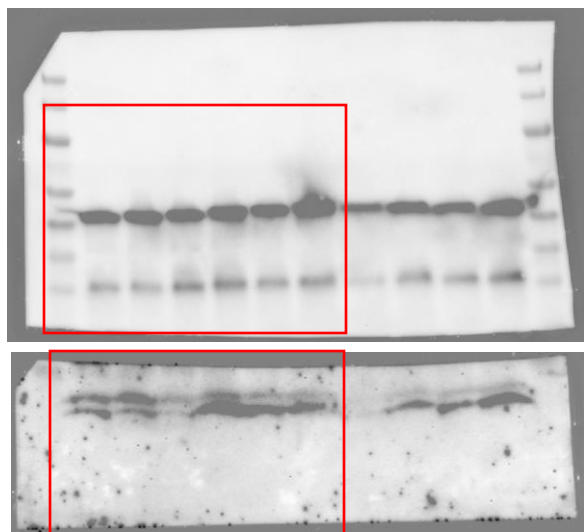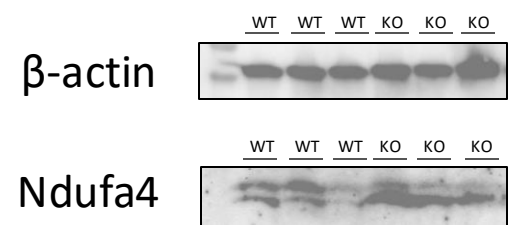

Supplement: Unedited blot and gel images [file jciinsight-10-190466-s074.pdf]
